# Qualitative evidence regarding the experience of receiving and providing care for mental health conditions in non-specialist settings in low-income and middle-income countries: a systematic review of reviews

**DOI:** 10.1136/bmjment-2023-300755

**Published:** 2023-08-23

**Authors:** Petra C Gronholm, Akerke Makhmud, Corrado Barbui, Elaine Brohan, Neerja Chowdhary

**Affiliations:** 1 Health Service and Population Research Department, Institute of Psychiatry, Psychology and Neuroscience, King's College London, London, UK; 2 WHO Collaborating Centre for Research and Training in Mental Health and Service Evaluation, Department of Neuroscience, Biomedicine, and Movement Sciences, Section of Psychiatry, University of Verona, Verona, Italy; 3 Department of Mental Health and Substance Use, World Health Organization, Geneva, Switzerland

**Keywords:** PSYCHIATRY

## Abstract

**Question:**

This review of reviews synthesises qualitative evidence on the experiences of receiving and providing care and treatment for mental health conditions in non-specialist settings in low-income and middle-income countries (LMICs), and the factors that influence the provision and uptake of such services.

**Study selection and analysis:**

Database searches were conducted in PubMed/MEDLINE, EMBASE, PsycINFO, CINAHL, Scopus, African Index Medicus and Global Index Medicus, supplemented by screening repositories of systematic reviews protocols and contacting authors. The evidence synthesis drew on deductive and inductive approaches: a framework analysis approach was used for the initial coding structure, after which the results synthesis was refined further through reviewing and regrouping the initial coding through thematic synthesis principles.

**Findings:**

Nine reviews met inclusion criteria and reported on a range of factors related to the provision and uptake of mental healthcare by non-specialist health workers in LMICs: (1) health worker competency, (2) availability of resources, (3) recipient-related and provider-related characteristics, (4) service accessibility, (5) sociocultural acceptability and (6) vulnerable groups for whom barrier to care were potentially exacerbated.

**Conclusions:**

This review provides nuanced and contextualised insights regarding the experiences of receiving and providing care for mental health conditions in LMICs, including barriers influencing service provision and uptake. It is important to ensure mental healthcare in non-specialist settings in LMICs is delivered in a manner which is feasible, acceptable and culturally appropriate in order to improve access to care, reducing stigma and promoting better overall health and well-being for individuals and communities.

**WHAT IS ALREADY KNOWN ON THIS TOPIC**

- Studies on the experience of receiving and providing mental healthcare in non-specialist settings in low-income and middle-income settings (LMICs) have reported fragmented and at times contradictory results.

**WHAT THIS STUDY ADDS**

- This review of reviews examined qualitative evidence on experiences of care for mental health conditions in non-specialist settings in low-income and middle-income countries (LMICs).
- The synthesised results emphasised a number of factors related to service uptake and provision, reflecting: health worker competency, availability of resources, recipient-related and provider-related characteristics, service accessibility, sociocultural acceptability and vulnerable groups.

**HOW THIS STUDY MIGHT AFFECT RESEARCH, PRACTICE OR POLICY**

- These results provide nuanced and contextualised insights regarding the experiences of receiving and providing care for mental health conditions in LMICs, which can inform and improve programmes, interventions and policies aiming to facilitate the provision of care for mental health conditions in low-resource settings.
- Insights from this study could enrich, for example, the evidence used to inform the WHO Mental Health Gap Action Programme recommendations.

## Background

There is increasing attention to the provision of care and treatment of mental health conditions in low-income and middle-income countries (LMICs). Mental health conditions, that is, mental, neurological and substance use disorders, cause a significant global burden of disease,^1^ and can have significant social and economic impacts on individuals and communities. By improving access to care, we can mitigate these impacts and promote better overall health and well-being. Mental health services are often underfunded and inaccessible, particularly in LMICs compared with high-income countries.^2–4^ This contributes to the gap between need and provision of services for such conditions^5^—a global issue which is exacerbated in low-resource settings.^6 7^

The WHO recommends addressing this issue through providing assessment and management of mental health conditions within non-specialist services settings. This is particularly important in LMICs, where availability of and access to specialist care services is scarce. For example, the Mental Health Gap Action Programme (mhGAP)^8^ has been developed specifically to support the scaling up of care for mental health conditions in low-resource settings. It has a specific focus on defined priority mental, neurological and substance use conditions, selected due to their high public health burden in terms of mortality, morbidity and disability; large economic costs and association with human rights violations.^9^

Quantitative evidence on the effectiveness of the provision of care for mental health conditions in non-specialist low-resource settings has been synthesised previously. For example, trial-based data show that interventions led by community‐based primary health workers in LMICs improved outcome for a range of mental health conditions, such as common mental conditions and substance use.^10^ However, despite the fact that there are effective treatments, a gap in access/provision still remains. To understand this further and improve service uptake and utilisation, it is important to consider the perspectives of those providing and using mental health services in non-specialist settings in LMICs. Although there are past qualitative studies and systematic reviews on this topic, their overall results have been fragmented and at times contradictory. A consolidated understanding of the experience of receiving and providing care and treatment would facilitate identifying the gaps and challenges in current service delivery systems and work to improve provision of and access to care.

## Objective

Given this, the objective of this review is to synthesise qualitative evidence on the experiences of receiving and providing care and treatment for mental health conditions in non-specialist settings in LMICs, and the factors that influence the provision and uptake of such services.

### Study selection and analysis

This review is a systematic review of reviews. The review protocol was registered on PROSPERO (CRD42022315291), and the review adheres to Preferred Reporting Items for Systematic Reviews and Meta-Analyses reporting guidelines.

### Search strategy and eligibility criteria

This review included reviews that synthesised qualitative evidence on the experiences of receiving or providing care and treatment for mental health conditions in non-specialist health settings in LMICs, and factors influencing the uptake/provision of such services. The search strategy was structured around terms reflecting: population (service users/caregivers/providers) AND intervention (care for mental health conditions in non-specialist settings) AND context (LMICs) AND outcome (service uptake, experience/provision of care; barriers/facilitators to care uptake/provision) AND qualitative research AND systematic reviews. There were no restrictions on publication language or date. Table 1 outlines the eligibility criteria for these terms, see online supplemental appendix 1 for the full search strategy.

10.1136/bmjment-2023-300755.supp1 Supplementary data

**Table 1:** Review eligibility criteria

Population	Include: People using/receiving care for mental health conditions in non-specialist settings (eg, service users, patients, clients, stakeholders, caregivers) people providing care for mental health conditions in non-specialist settings: healthcare providers in primary care settings (eg, healthcare worker, health staff, health professional, medical staff) Exclude: People using/receiving care in specialist mental health settings or for physical health conditions. Specialist mental healthcare providers (eg, mental health nurses, specialist counsellors, psychologist, psychiatrists)
Intervention	Include: Care for mental health conditions in non-specialist settings. Specifically, the review included mental, neurological and substance-use conditions that are priority conditions addressed by mhGAP (ie, depression, psychosis and bipolar disorder, suicide and self-harm; epilepsy and seizures; dementia; alcohol use disorders, drug use disorders; child and adolescent mental health disorders (autism and other developmental disabilities); conditions related to stress; anxiety) or broader descriptions of these conditions (eg, ‘mental health conditions’) Exclude: care for mental, neurological and substance-use conditions provided in specialist settings (eg, decidated mental health services/clinics); care for mental health conditions beyond the mhGAP priority conditions outlined above; care for physical health conditions.
Outcome	Include: Processes, views and experiences reflecting uptake and/or provision of care (eg, patient compliance, delivery of healthcare, utilisation) Factors reflecting barriers/facilitators influencing service uptake and/or care provision (eg, patient acceptance of healthcare, health services accessibility, availability) Exclude: Quantitative assessments of these processes and influences. Clinical or prevalence-related aspects of service uptake and/or care provision; for example, symptom severity or reduction, clinical effectiveness, screening rates, service utisation rates, compliance rates, treatment coverage.
Context	Include: Low-income and middle-income countries (LMICs), as specified using the Cochrane LMIC 2020 filter* Exclude: High-income countries; countries not classified as LMICs by the Cochrane LMIC 2020 filter.
Type of studies	Include: Peer-reviewed systematic reviews focused on qualitative evidence, specifically: qualitative evidence; qualitative narrative results syntheses of evidence; mixed-methods evidence reported in a qualitative narrative manner. Exclude: Systematic reviews synthesising purely quantitative evidence, non-peer reviewed systematic reviews, non-systematic reviews (ie, narrative reviews, commentaries), studies focused on primary data.

*https://epoc.cochrane.org/sites/epoc.cochrane.org/files/public/uploads/epoc_lmic_filters_2020_v4.docx (accessed 1 March 2022).

mhGAP, Mental Health Gap Action Programme.

The searches were conducted in March 2022 in the following databases: PubMed/MEDLINE, EMBASE, PsycINFO, CINAHL, Scopus, African Index Medicus, Global Index Medicus (Index Medicus for the Eastern Mediterranean Region, Index Medicus for the South-East Asian Region, Latin American and Caribbean Health Sciences Literature and Western Pacific Region Index Medicus). These database searches were supplemented by sourcing potentially relevant reviews for inclusion through screening repositories of systematic reviews protocols (PROSPERO, Open Science Framework and Cochrane), and contacting authors of potentially relevant protocols to hear if their work had been published and could be screened for relevance for inclusion this review of reviews.

### Study selection

Search results were initially screened based on their title and abstracts (English abstracts were available for screening all articles), followed by full text screening of the articles deemed potentially relevant for inclusion. For articles published in languages in other than English, Google Translate (with added input by a native language speaker) was used to translate the full texts for screening. Two authors (PCG and AM) independently screened the first 20% of the records at both stages. Discrepancies were resolved through discussion, with a third author (NC) serving as arbitrator if needed. Once consistency in screening was achieved, the remaining records were shared for screening between the two authors.

### Data extraction and synthesis

Data from eligible articles were extracted into a predefined template capturing review design, methods, setting, population, intervention details and relevant outcomes (service uptake, experience/provision of care, barriers/facilitators to care uptake/provision). For outcomes, relevant data for extraction and subsequent analysis were review findings labelled as ‘results’ or ‘findings’, as reported in the reviews’ results, discussion and abstract sections.

To minimise errors in data extraction, the data extraction template was piloted by two authors (PCG and AM) using an example review prior to the main data extraction, after which the extraction template headings were refined for clarity. The data extraction process was shared between two authors (PCG and AM). Each author extracted details from half of the included studies, after which the other author reviewed the extracted data, followed by a discussion of the process (clarification and consolidation of potential discrepant views) to reach final agreement on the extracted data.

The evidence synthesis drew on both deductive and inductive approaches. A framework analysis approach^11^ was used as an initial guide for the coding structure, based on domains in the Grading of Recommendations Assessment, Development and Evaluation Evidence to Decision framework^12^ (eg, considerations of health equity, equality and non-discrimination related to service receipt or provision, feasibility of service uptake/delivery, sociocultural acceptability of interventions). This initial deductively driven grouping was refined further through an inductive approach drawing on thematic synthesis principles.^13^ This involved building on the initial coding structure through examining the data groupings, and developing these further through pruning and grouping in an axial coding process to develop analytical themes reflecting inductive, thematically coherent concepts in the data.

### Quality appraisal

We assessed the methodological quality of the included reviews using the Checklist for Systematic Reviews by the Joanna Briggs Institute (JBI).^14^ This critical appraisal checklist is specifically developed for systematic reviews of reviews and is suitable for assessing qualitative systematic reviews. The critical appraisal of the included reviews was conducted independently by two reviewers (PCG and AM); discrepancies were resolved through the same approach as was applied during the study selection process to establish final agreed ratings.

### Findings

**Figure 1:**
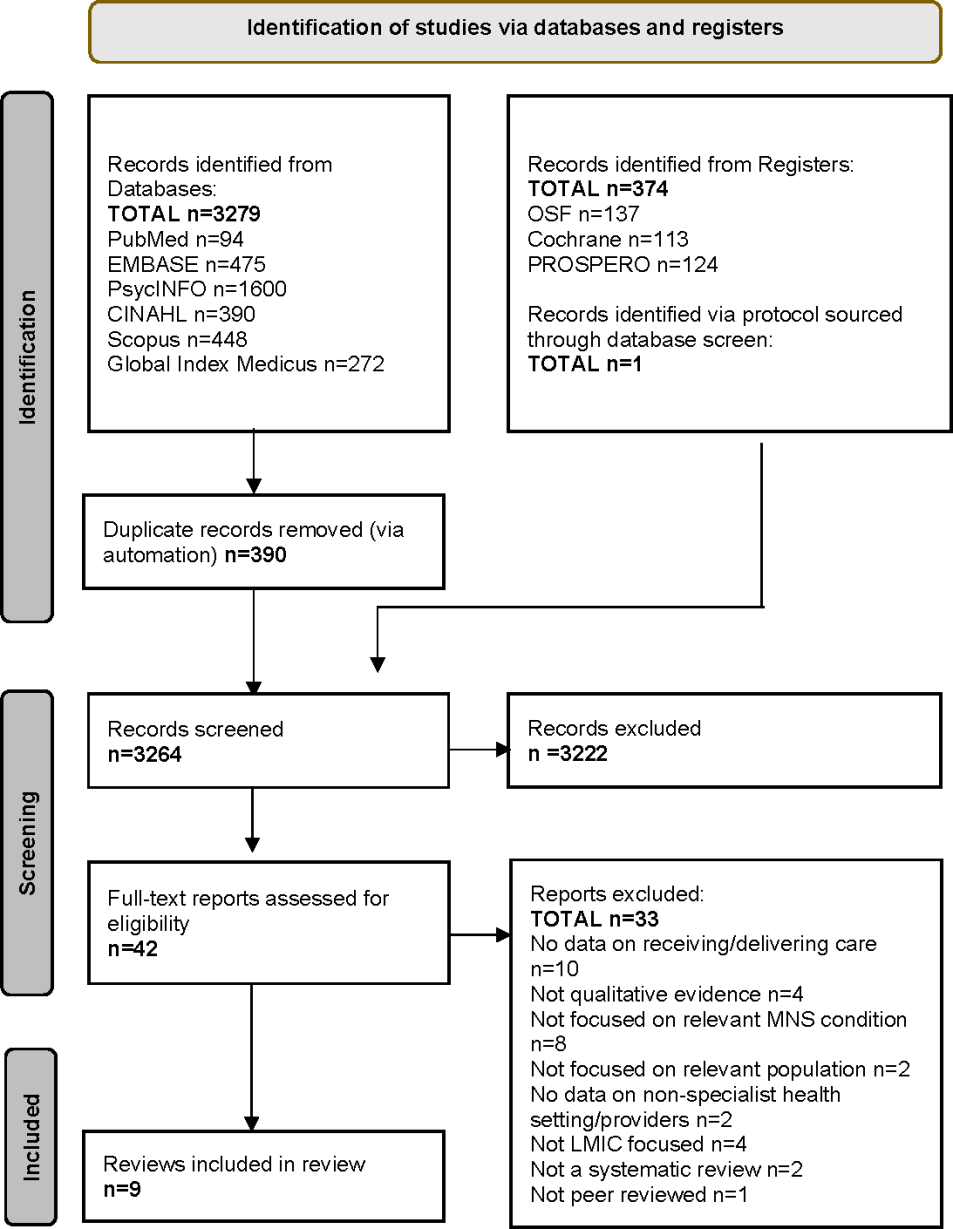
PRISMA flow diagram of study selection process.

The search produced 3264 records after removing duplicates, of which 42 were considered for full-text screening. Nine studies met review inclusion criteria; figure 1 illustrates this study selection process.

Table 2 details the design and methods of the included reviews, and table 3 provides an overview of their focus in terms of key domains of interest (population (service users and healthcare providers), healthcare intervention, mental health condition). In brief, most were mixed-methods reviews (n=7)^15–21^ whereas one considered qualitative, quantitative and mixed-methods evidence separately^22^ and one was focused on qualitative evidence only.^23^ In terms of setting in focus, four reviews focused on LMICs generally.^17 19–21^ Two articles had a country-specific focus,^15 16^ and the remaining publications focused on a specified range of countries (eg, sub-Saharan Africa).^18^ In terms of mental health condition, most reviews focused on mental health conditions generally (n=7)^15 16 18 19 21–23^ and two reviews focused specifically on schizophrenia^17^ and substance use.^20^

**Table 2:** Included reviews—design and methods

First author, year	Review aims	Description of review approach	Articles (studies) included	Qualitative data collection approach in included studies	Description of data synthesis
Amaral,^15^ 2018	Explore the characteristics of pathways to mental healthcare in Brazil, synthesising evidence from published quantitative and qualitative research. Specific objective: to articulate the results with different national mental health policies adopted over time and to highlight evidence for each pathway stage.	Systematic review; mixed methods	25	Individual interviews, focus groups, participant observation	Narrative synthesis
Badu,^16^2018	1.To identify the existing enablers facilitating access to psychiatric services. 2. To synthesise the existing barriers confronting mental health service users to accessing mental health services.	Integrative review; mixed methods	42	Document reviews, field observations, focus groups, individual interviews	Mixed-methods synthesis, quantitative and qualitative data assimilated into a single synthesis and content analysis applied to derive commonalities
Brooke-Sumner,^17^ 2015	To systematically assess the evidence for feasibility and acceptability of community-based psychosocial interventions for schizophrenia in LMICs and to generate recommendations for practice and priorities for future research.	Systematic review; mixed methods	17	Individual interviews, written and verbal accounts of patients’ perceptions of intervention; focus groups	A qualitative (thematic) synthesis of qualitative and quantitative data
Mutahi,^18^ 2022	To synthesise evidence about the mental health problems, methodologies and service delivery gaps experienced by pregnant adolescent girls and young women in sub-Saharan Africa.	Systematic review; mixed methods	18	Individual interviews, focus groups	(No clear statement provided)
Padmanathan,^19^ 2019	To summarises current findings and highlights barriers which task-sharing will need to overcome if it is to be scaled up as a strategy to reduce the treatment gap for mental disorders.	Systematic review; mixed methods	17 (21)	Individual interviews, verbally administered qualitative questionnaires, focus groups, observational sessions	Comparative thematic approach of qualitative and quantitative data
Ryan,^22^ 2021	To investigate key characteristics of the interventions tested, the methods used to evaluate them, and the evidence generated to date, to describe the current state of the research in this area.	Systematic review; qualitative, quantitative and mixed methods	23	Individual interviews, focus groups	Adapted narrative synthesis
Sarkar,^20^ 2021	Look at the barriers and facilitators of substance use disorder treatment in LMICs.	Qualitative review synthesis; mixed methods	28	Focus groups, field observations, individual interviews, ‘qualitative’ methods	(No clear statement provided)
Verhey,^21^ 2020	Primary objectives: (1) better understand how CBT-based interventions are delivered by non-specialist health workers by considering implementation outcomes; (2) include pilot, feasibility and qualitative studies in addition to RCTs to gather key implementation information and (3) consider the treatment of substance-use disorder in LMICs with CBT. Secondary objectives: (1) to explore implementation outcomes by provider type; (2) examine provider-level factors facilitate implementation and (3) identify how training and supervision strategies support implementation.	Systematic review; mixed methods	18	Individual interviews, focus groups	Narrative synthesis
Dickson,^23^ 2018	To synthesise evidence from studies of people’s experiences and perspective of mental health and psychosocial support programmes from our qualitative evidence on the barriers to and facilitators of, implementing and receiving such programmes delivered to populations affected by humanitarian emergencies in LMICs	Systematic review; qualitative (included mixed-methods paper but considered qualitative evidence)	15 (14)	Document review; individual interviews; focus groups, clinical field observations, participatory research workshops; curriculum feedback activities, evaluation forms	Narrative thematic synthesis

CBT, cognitive behavioural therapy; LMICs, low-income and middle-income countries; RCTs, randomised controlled trials.

**Table 3:** Included reviews—key domains of interest

First author, year	Setting	Service users*	Healthcare providers†	Care/treatment/intervention	Mental health condition
Amaral,^15^	Brazil	People of any age seeking help	Primary healthcare workers, GP and other general health services, primary care professionals.	Experience of help-seeking from primary care, Recognition of mental health problem (by GP or other non-specialist), decision to treat or refer (by GP/in primary care)	Mental health problems, ‘health-related distress’
Badu,^16^	Ghana and ‘multicountry studies including Ghana’	Mental health service users, family members and caregivers	Service providers (non-specialists)	Mental health services at non-specialist level	General mental health issues
Brooke-Sumner,^17^	LMICs	General adult population (who need treatment for schizophrenia)	Community lay health workers/non-specialist health workers (delivering community-based psychosocial interventions to people with schizophrenia or their caregivers)	Community-based psychosocial interventions to people with schizophrenia or their caregivers	Schizophrenia
Mutahi,^18^	Sub-Saharan Africa (Nigeria, Kenya, South Africa, Malawi, Uganda)	Pregnant adolescent girls and young women 12–24 years old with mental health needs	Healthcare setting: maternal and child health clinics in primary care or public health services, healthcare providers, healthcare workers from maternal and child health clinics, community health workers,	Healthcare settings (most commonly maternal and child health clinics in primary care or public health services)	Mental health problems
Padmanathan,^19^	LMICs	‘Service users’; all ages, both genders, families, caregivers.	Task-sharing personnel: community health workers, paraprofessionals, non-specialists nurses, paramedics, social workers, medical officers, recovered service users.	Primary healthcare or community-based task-sharing interventions to identify or diagnose mental conditions or improve the mental health	Mental disorders, mental health
Ryan,^22^	Tanzania, Uganda, Zimbabwe, Pakistan, Thailand, Colombia	Adults who have first-hand experience of a humanitarian crisis that occurred during their lifetime	Lay health workers, primary care workers, lay counsellors, nurses or medics working in primary health centres.	Evidence-based talk therapies delivered through in-person dialogue with a trained lay worker, either one-to-one or in a group format, for the treatment of common mental disorders	Common mental disorders (including no confirmed diagnosis, subthreshold)
Sarkar,^20^ 2021	LMICs	Patients receiving treatment for substance use disorders	Not specified (non-specialists)	Community-based substance use treatments	Substance use
Verhey,^21^ 2020	LMICs	Not specified	Non-specialist health workers	Cognitive behavioural therapy-based intervention	Common mental disorders or substance use disorders
Dickson,^23^ 2018	‘Humanitarian crises regions’‡	Adults or children affected by humanitarian emergencies	‘Programme providers’ (non-specialists)	Mental health and psychosocial support programmes	Psychosocial well-being and/or prevent or treat mental disorder

*Treatment receving population (service users).

†Treatment providing group/population (healthcare workers).

‡Northern Uganda, Haiti, Sierra Leone, Rwanda, Mozambique, Iran, Palestina/Israel, Sri Lanka, Turkey, Burundi.

GP, general practitioner; LMICs, low-income and middle-income countries.

In terms of methodological quality, the included reviews were scoring moderately to highly on the JBI quality appraisal tool; see table 4 for overview of scores. The quality domains in which most reviews lacked was whether the likelihood of publication bias was assessed, whether the critical appraisal of articles included in the review was conducted by two or more reviewers independently, and whether there were methods used to minimise errors in data extraction. All included reviews met the criteria of a clear and explicitly stated review question, appropriate inclusion criteria, appropriate quality appraisal criteria and appropriate directives for new research.

**Table 4:** Scores on the quality appraisal checklist for systematic reviews by the Joanna Briggs Institute

	Quality appraisal checklist item* (Y=yes, criteria met; N=no, criteria not met; U=unclear; n/a=not applicable)											Total	% of 11
	1	2	3	4	5	6	7	8	9	10	11		
Amaral *et al*,^15^ 2018	Y	Y	Y	Y	Y	U	N	Y	N	n/a	n/a	6	55
Badu *et al*,^16^ 2018	Y	Y	Y	Y	Y	Y	U	Y	N	Y	Y	9	82
Brooke-Sumner *et a*l,^17^ 2015	Y	Y	Y	Y	Y	U	Y	Y	N	Y	Y	9	82
Mutahi *et al*,^18^ 2022	Y	Y	Y	Y	Y	Y	Y	Y	U	Y	Y	10	91
Padmanathan and De Silva,^19^ 2013	Y	Y	Y	Y	Y	U	N	Y	N	Y	Y	8	73
Ryan *et a*l,^22^2021	Y	Y	Y	Y	Y	U	Y	Y	N	N	Y	8	73
Sarkar *et a*l,^20^ 2021	Y	Y	Y	N	Y	U	N	U	N	Y	n/a	5	45
Verhey *et al*,^21^ 2020	Y	Y	Y	Y	Y	Y	N	Y	N	Y	Y	9	82
Dickson and Bangpan,^23^ 2018	Y	Y	U	Y	Y	N	Y	Y	N	Y	Y	8	73

*(1) Is the review question clearly and explicitly stated?; (2) Were the inclusion criteria appropriate for the review question?; (3) Was the search strategy appropriate?; (4) Were the sources and resources used to search for studies adequate?; (5) Were the criteria for appraising studies appropriate?; (6) Was critical appraisal conducted by two or more reviewers independently?; (7) Were there methods to minimise errors in data extraction?; (8) Were the methods used to combine studies appropriate?; (9) Was the likelihood of publication bias assessed?; (10) Were recommendations for policy and/or practice supported by the reported data?; (11) Were the specific directives for new research appropriate?

No articles were excluded based on their quality rating, but the quality assessment was considered in the results synthesis. When synthesising the findings, insights from the articles with the weakest methodological quality ratings^15 20^ were not highlighted as singular source for any conclusions, but rather were included as examples alongside evidence sourced from other articles of more robust methodological quality.

The included reviews reported on a range of experiences and influencing factors related to the provision and uptake of mental healthcare by non-specialist health workers in LMICs. These findings are synthesised in the following six themes: (1) health worker competency, (2) availability of resources, (3) recipient-related and provider-related characteristics, (4) service accessibility, (5) sociocultural acceptability and (6) vulnerable groups for whom barrier to care were potentially exacerbated.

#### Health worker competency

The competency and work experience of healthcare workers was highlighted as one factor influencing mental health intervention delivery. Self-perceived competency (eg, ability to deliver intervention, communicate core concepts, counselling competency, remembering guidelines, developing treatment plans) is one part of the healthcare worker competency that comes into play.^19^ Competency from an external perspective was also a factor, including scepticism or negativity towards task-sharing workforce by other healthcare workers, non-specialist mental health personnel not being regarded as an important part of the healthcare system, and health system managers unwilling to prioritise counselling due to lack of understanding.^19^ Challenges related to competency were suggested as mitigated through training, refreshers, supervision and networking with others in same role.^19^

#### Availability of resources

The availability of resources was a further key influencing factor for the provision and uptake of mental healthcare. The impact was present on several levels. On a structural level, the lack of investment for mental health was discussed as a challenge to planning and delivering mental health services at primary care level.^16^ On a health-systems level, a lack of funding also impacted infrastructure, such as the availability of transport for home visits, or suitable locations for service delivery.^19^ Additionally, a lack of funding could result in barriers to providing training and supervision, which is particularly important for task-sharing interventions involving non-specialist staff, and the sustainability of these efforts.^22 23^ Limited resources also in terms of specialist staff can be a challenge, if interventions with non-specialist health workers also require specialist staff to prescribe medications and supervise treatment.^22^ It was suggested health worker resource availability could be improved by minimising the burden of their involvement with further interventions and programmes, for example, through integrating contributions to non-specialist interventions into existing commitments (eg, ongoing training), or assigning different staff different duties.^19^ Limited availability of the non-specialist healthcare work force was also noted, with possible barriers to intervention feasibility including shortages of counsellors, scarcity of people for task-sharing roles, availability of suitable people (eg, literacy requirements), high cadre turnover and competing responsibilities (eg, family).^19^ At the caregiver level, intervention feasibility and sustainability were reported to be influenced by caregivers’ ability to join due to employment, family members’ inability to join home-based programmes or family workshops, inconvenience for family members to attend group sessions due to lack of interest or other responsibilities (eg, childcare), and unsuitable session timing.^17^ Availability of personal financial resources was another participant-level factor, with costs (affordability of medication and treatment) a potential barrier to access/service engagement.^20^

In terms of resources, there were also some indications of task-sharing interventions reducing costs: two examples using trained and supervised lay health workers (non-medical personnel instead of health workers) reduced the implementation cost of psychoeducation programmes, which made their implementation more feasible.^17 21^

#### Recipient-related and provider-related characteristics

Recipient-related characteristics could also affect the engagement with treatment for mental health conditions. For example, participants’ education and literacy levels can affect how much the participants are able to engage with the intervention if it involves writing-based tasks, or information and instructions inappropriate for the literacy levels of the care receivers or their caregivers.^17^ Other factors related to the care providers, and included the perceived safety risks for service providers doing home-visits (especially regarding the schizophrenia intervention),^17^ lack of private office/space to carry out intervention, and restrictions on abilities to prescribe medication.^19^ Additionally, reaching the eligible participants or getting the buy-in of carers to sustain the participation in treatment^23^ could be another barrier for the service providers.

#### Service accessibility

Practical considerations of service accessibility were another key factor in intervention uptake and delivery. Travel related barriers (eg, time and cost),^19^ affordability of travel to receive care, reaching/retaining participants who are very mobile (eg, moving out of district/family home),^17^ challenges to make time due to demands of occupation (eg, causal work, daily pay),^20^ and inconvenient hours of operation^15^ could all act as barriers both for lay healthcare providers and service recipients. Informational accessibility was identified as another important factor. Limited awareness about existing service facilities could contribute to the lack of accessibility.^16^ Additionally low ability to impact potential recipients’ interest,^20^ lack of motivation^21^ and perceived levels of usefulness of the interventions could result in additional challenges.^17^ Similarly, negative perceptions of help could be a barrier.^18^ It was mentioned that these barriers could be overcome through providing mental health services and screening within primary care settings also to reduce stigma and raise awareness.^18^

#### Sociocultural acceptability

When considering factors influencing service provision and uptake, the importance of sociocultural acceptability of mental health programmes was clearly expressed. For example, inadequate considerations of the cross-cultural applicability of how services were provided could lead to lack of satisfaction in the treatments implemented.^19 22^ Also, when interventions had been sociocultural adapted, and when interventions were perceived as appropriate for the culture and target group, participants had a more favourable response to them and their content and medium of delivery received more positive feedback from service users and caregivers.^17 19 21 23^ Breaches to sociocultural acceptability were evident in reflections on stigma-related concerns and experiences: poor treatment and stigmatisation by health providers and confidentiality issues were a concern for access to the services.^17 18 20 21^ Beyond cultural sensitivity, considerations of appropriateness regarding age, sex and language were highlighted as important to increased intervention acceptability and accessibility.^18 19 21 22^

#### Vulnerable groups

It was reported that the factors influencing service uptake and specifically barriers to care were exacerbated among some groups of potential recipients. The main groups affected included women, people with low levels of education and literacy, or people from low resource settings. Women were reported to face particular access challenges^17 20^ through their inability to travel away from their own locality, and stronger concerns regarding stigma and shame (noted for substance use particularly). This disparity might also be particularly pronounced for caregiver interventions, where women are less often able to participate due to other caring responsibilities.^17^ Another vulnerable group was people with low levels of education or literacy^17^ who might struggle with writing-based tasks and taking instructions compromising the utility of psychoeducation materials. Lastly, people from low-resource settings were considered to be particularly impacted by affordability and cost challenges.^20 22 23^

## Conclusions and clinical implications

This review examined qualitative evidence on receiving and delivering care for mental health conditions in non-specialist settings in LMICs. The reviews considered in this synthesis emphasised a number of factors related to service uptake and provision, detailing barriers to these processes. These barriers influencing service provision and uptake could be considered to contribute health equity, equality and non-discrimination, as well as impacting people’s right to health and access to healthcare. Achieving the benefits of mental health interventions delivered by non-specialist health workers are dependent on whether the programmes are feasible and acceptable, and it thus important to consider how related barriers can be mitigated.

Addressing stigma around mental health in communities and addressing the sociocultural acceptability of programmes would play a role in the uptake and delivery of the mental health interventions in LMICs. For example, stigma and discrimination could affect help-seeking, and lack of confidentiality could deter people from accessing care or receiving confidential and safe mental healthcare. Such potential barriers can result in people not getting the needed quality treatment.

Also, the findings regarding the perceived cultural and local appropriateness of care provision point to the importance of adapting interventions, including through considering service user perspectives and sociocultural differences, to ensure interventions are meaningful for service users in a given context. Considering the wider context is critical when delivering mental healthcare, particularly in LMIC settings.^24^ Potential mitigating steps to improve sociocultural acceptability could include training health workers in non-judgemental care, integrating preventative mental health awareness messages to reduce the stigma and training acceptable counsellors for the local settings and target groups as well as facilitating the use of indigenous/local phrases and terms to increase treatment acceptability, accessibility and fidelity.

The findings of this review also highlight the need to provide appropriate funding and resources for service provision, from a structural level to investment in services and also considering resources at the individual level.

In considering the articles included in this review, apparent gaps in this qualitative evidence based can be highlighted to guide future research. In terms of the available evidence, it should be noted that the included review articles generally pooled data from a cross a range of different non-specialist health service providers (eg, primary healthcare workers, general practitioners, and other general health services and primary care professionals^15^), or without providing details on the characteristics of the personnel in question.^16^ Most articles also considered a range of mental health conditions without providing details of the specific reasons for uptake and/or provision of services.^15 16 18 19 21–23^ This general nature of the evidence prevents the generation of specific insights that would further support the efforts to consider challenges and facilitators present for given circumstances. It is also likely that there are particular contextual differences between different settings—it should not be assumed that barriers and facilitators are comparable across different LMIC contexts. Future studies with a sharper focus would provide a more granular understanding, allowing for the development of targeted strategies to facilitate the process to obtain and provide care under specific conditions (depending on, eg, particular contextual or cultural setting, type of non-specialist service provider, intervention or mental health condition). Future work can also be informed by considering the methodological quality of evidence included in this review. The quality appraisal highlighted how, for example, it was unclear to which extent the included studies considered the impact of publication bias, involved two or more independent reviewers in the critical appraisal process, or used methods to minimise errors in data extraction. Future studies could aim to address these limitations and strengthen methodology of the research in this area, and produce sound systematic review evidence to inform future research and work related to mental healthcare provision. This could involve, for example, reflecting on dissemination bias^25^ and providing clearer reporting of methodological procedures. Another consideration is the tendency for reviews to consider only peer-reviewed evidence, and the risk of publication bias this introduces. This could be addressed through endeavouring to capture insights also from grey literature and unpublished work in future studies. This approach would reflect a more comprehensive and balanced range of work in the area, and potentially also enable more timely access to insights from research made available outside the academic publishing context. However, this broader focus would likely also introduce further heterogeneity and methodological variability into the results under consideration, warranting careful consideration when interpreting the findings and making recommendations based on these.

### Limitations

These findings also need to be considered in view of limitations inherent to this review. This review was conducted following a comprehensive search strategy across multiple databases. However, some relevant reviews might still not have been identified through the search. Other potentially relevant reviews for inclusion might also have been published since our searches were conducted. The studies included in this review do, however, provide a broad range of relevant evidence that allow us to draw useful conclusions regarding factors influencing engagement with and provision of mental health services as relevant for this review. Also, as only 20% of the records were independently screened by two authors, it is possible that this introduced some bias to the study selection process. Inter-rater reliabilities were not calculated for the quality appraisal scores prior to these being consolidated through discussion, limiting the ability to assess for potential bias in the process. It is recognised that through excluding non-peer-reviewed articles from the review, there is an increased risk of publication bias influencing the results (a risk of publication bias was also noted when considering the articles included in the review). However, this choice ensured all included materials had undergone a rigorous peer-review process, intended to ensure that the included research represents scientifically robust data. The heterogeneity of the included articles means that specific contextual nuances in experiences cannot be fully captured, and the conclusions drawn remain general in nature. Also, some aspects of the results rely on a limited number of sources, such as the ‘health worker competency’ results theme,^19^ or findings related to specific diagnoses (schizophrenia,^17^ substance use^20^), which were all in focus in only one included article, respectively. This does limit the generalisability of the findings, however, as the included articles are reviews drawing on a single source still represents data from a number of studies.

## Conclusions

The results of this review can help provide more nuanced and contextualised insights regarding the experiences of receiving and providing care for mental health conditions in LMICs, which can serve to further inform and improve programmes such as mhGAP,^8^ and other interventions and policies aiming to facilitate the provision of care for mental health conditions, particularly in low-resource settings. For example, the recommendations provided by mhGAP are evidence based, backed by a rigorous process of collating and evaluating relevant evidence. To date, however, this process has been based on evidence from randomised controlled trials specifically, which means experiential perspectives of service provision and utilisation have been lacking. Considering also these kinds of insights alongside the clinical evidence of effectiveness would ensure that guidance regarding mental healthcare provided in non-specialist settings in LMICs is not only clinically effective, but also delivered in a manner which is feasible, acceptable and culturally appropriate. This is crucial for improving access to care, reducing stigma and promoting better overall health and well-being for individuals and communities.

## Data Availability

Data are available on reasonable request.
