# Supplementary material for: Qualitative evidence regarding the experience of receiving and providing care for mental health conditions in non-specialist settings in low-income and middle-income countries: a systematic review of reviews
Source: BMJ Ment Health. 2023 Aug 23;26(1):e300755. doi: 10.1136/bmjment-2023-300755 (PMC10577758; doi:10.1136/bmjment-2023-300755)
Supplement: Supplementary data [file bmjment-2023-300755supp001.pdf]

Online Appendix 1 – full search strategy

Search terms used to identify systematic reviews

Population (MeSH terms OR key words) AND Intervention (MeSH terms OR key words) AND Context (key words) AND Outcome (MeSH terms OR key words) AND Research type (key words)

+ "review" filter

PubMed

Detailed overview of search strategy

**Domain**	**Search terms (MeSH term and/or Key words)**	**Results 12/02/22**
**Population**	(service user OR caregiver OR health personnel[MeSH Major Topic])	497,237
	OR	
	service user* OR consumer* OR patient* OR stakeholder* OR user* OR client* OR carer* OR caregiver* OR parent* OR family OR relative* OR guardian* OR Health worker OR Health care worker OR Healthcare worker OR Health Care Provider* OR Healthcare Provider OR Health professional OR Health Care Professional OR Healthcare Professional OR Health staff OR Medical staff OR clinical staff OR medical workforce OR medical work force OR health workforce OR health work force	9,812,662 title/abstract
	AND	
**Intervention** *Care for mental health conditions*	Mental Disorders/prevention and control OR Nervous System Diseases/prevention and control OR Substance-Related Disorders/prevention and control OR Epilepsy/prevention and control OR Mental Health Services[MeSH Major Topic])	231,234
	OR	
	Mental Health Service OR Mental Healthcare OR Mental Health Care OR Mental Health System OR Psychiatric Services OR Psychiatric Care OR Psychiatric Health Care OR Psychiatric Healthcare OR Mental Illness OR Mental Health OR Severe Mental Disorder OR Common Mental Disorder OR	1,454,601 title/abstract

	Alcohol Use Disorder OR Alcohol Abuse OR Alcohol-Related Disorder OR Alcohol Related Disorder OR Alcohol Addiction OR<br><br>Substance Disorder OR Substance Abuse OR Substance-Use Disorder OR Substance Use Disorder OR<br><br>Opioid Abuse OR Opiate Addiction OR Opioid-Related Disorder OR Opioid Related Disorder OR Cannabis-Related Disorder OR Cannabis Related Disorder OR Cocaine Related Disorder OR Cocaine Related Disorder OR Cocaine Addiction OR Amphetamine-Related Disorder OR Amphetamine Related Disorder OR Amphetamine Addiction OR Heroin Dependence OR Heroin Abuse OR Heroin Addiction OR Substance Induced Psychos OR Substance-Induced Psychos OR<br><br>Depression OR Depressive Disorder OR Mood Disorder OR Major Depressive Disorder OR<br><br>Attention Deficit Disorder OR attention deficit hyperactivity disorder OR ADHD OR Conduct Disorder OR Neurocognitive Disorder OR Neurodevelopmental Disorder OR Neurodevelopmental disabilit* OR Developmental Disabilit* OR Developmental Disorder OR<br><br>Autism Spectrum Disorder OR Autistic Spectrum Disorder OR Aspergers Disease OR Aspergers Syndrome OR<br><br>learning disabilit* OR intellectual disabilit* OR intellectual development OR mental retardation OR hyperkinetic disorder OR tic disorder OR<br><br>self-harm* OR self-injur* OR suicid* OR<br><br>Dementia OR Alzheimer* OR Parkinson* OR<br><br>Epilep* OR seizure OR<br><br>Schizophrenia OR Psychosis OR Psychoses OR Psychotic Disorder OR Schizoaffective Disorder OR Schizophreniform Disorder OR Psychotic Affective Disorder OR Psychotic Mood Disorder OR Affective Psychosis OR schizotypal OR delusional OR<br><br>Bipolar Disorder OR Manic Depressive Psychosis OR Bipolar Affective Psychosis OR Bipolar Affective Disorder OR Manic Depressive Psychoses OR Bipolar Depression OR Post-Traumatic Stress Disorder OR Post Traumatic Stress Disorder OR Traumatic Stress Disorder OR Stress Disorder OR acute stress reaction OR grief OR<br><br>Anxiety Disorder OR phobi* OR agoraphobi* OR panic disorder OR GAD OR obsessive compulsive disorder OR OCD	
	AND	

**Outcome**<br><br>views and experience of care *uptake* and/or care *provision;* factors (barriers/facilitators) influencing service *uptake* and/or care *provision*	Patient Acceptance of Health Care OR Delivery of Health Care OR Health Services Accessibility OR Patient Reported Outcome Measures OR Quality of Health Care OR Patient Compliance OR Healthcare Disparities OR Health Services[MeSH Major Topic]	8,776,041
	OR	
	facilitat* OR enhanc* OR enable* OR opportunity* OR encourag* OR motivat* OR promot* OR influen* OR<br><br>barrier* OR challenge* OR block* OR challeng* OR constrain* OR deter* OR difficult* OR discourag* OR disincentive* OR encumber* OR encumbranc* OR hinder* OR hindrance* OR impair* OR impede* OR impeding OR impediment* OR limit* OR delay OR obstruct* OR problem OR restrain* OR restrict* OR interfer* OR<br><br>perceive* OR perception* OR perspective* OR view* OR experience* OR need* OR attitude* OR belie* OR opinion* OR<br><br>quality OR Implementat* OR adoption OR<br><br>patient experience OR uptake* OR utilis* OR utiliz* OR<br><br>((use OR acceptance OR acceptability OR availability OR accessibility OR access OR accessing OR receipt OR receive OR received OR receiving) AND (health care OR healthcare OR patient care OR health service* OR primary care OR visit OR appointment)) OR<br><br>((provide OR providing OR provis* OR distribut* OR deliver*) AND (health care OR healthcare OR patient care OR health service* OR primary care OR visit OR appointment))	9,686,489 title/abstract
	AND	
**Context**<br><br>*LMICs*	afghanistan OR albania OR algeria OR american samoa OR angola OR antigua OR barbuda OR argentina OR armenia OR armenian OR aruba OR azerbaijan OR bahrain OR bangladesh OR barbados OR belarus OR byelarus OR belorussia OR byelorussian OR belize OR british honduras OR benin OR dahomey OR bhutan OR bolivia OR bosnia OR herzegovina OR botswana OR bechuanaland OR brazil OR brasil OR bulgaria OR burkina faso OR burkina fasso OR upper volta OR burundi OR urundi OR cabo verde OR cape verde OR cambodia OR kampuchea OR khmer republic OR cameroon OR cameron OR cameroun OR central african republic OR ubangi shari OR chad OR chile OR china OR colombia OR comoros OR comoro islands OR mayotte OR congo OR zaire OR costa rica OR cote d'ivoire OR cote d' ivoire OR cote divoire OR cote d ivoire OR ivory coast OR croatia OR cuba OR cyprus OR czech republic OR czechoslovakia OR djibouti OR french somaliland OR dominica OR dominican republic OR ecuador OR egypt OR united arab republic OR el salvador OR equatorial guinea OR spanish guinea OR eritrea OR estonia OR eswatini OR swaziland OR ethiopia OR fiji OR gabon	431,591 title/abstract

	OR gabonese republic OR gambia OR georgia OR georgian OR ghana OR gold coast OR gibraltar OR greece OR grenada OR guam OR guatemala OR guinea OR guyana OR guiana OR haiti OR hispaniola OR honduras OR hungary OR india OR indonesia OR timor OR iran OR iraq OR isle of man OR jamaica OR jordan OR kazakhstan OR kazakh OR kenya OR korea OR kosovo OR kyrgyzstan OR kirghizia OR kirgizstan OR kyrgyz republic OR kirghiz OR laos OR lao pdr OR lao people's democratic republic OR latvia OR lebanon OR lesotho OR basutoland OR liberia OR libya OR libyan arab jamahiriya OR lithuania OR macau OR macao OR macedonia OR madagascar OR malagasy republic OR malawi OR nyasaland OR malaysia OR maldives OR indian ocean OR mali OR malta OR micronesia OR kiribati OR marshall islands OR nauru OR northern mariana islands OR palau OR tuvalu OR mauritania OR mauritius OR mexico OR moldova OR moldovian OR mongolia OR montenegro OR morocco OR ifni OR mozambique OR portuguese east africa OR myanmar OR burma OR namibia OR nepal OR netherlands antilles OR nicaragua OR niger OR nigeria OR oman OR muscat OR pakistan OR panama OR papua new guinea OR paraguay OR peru OR philippines OR philipines OR phillipines OR phillippines OR poland OR polish people's republic OR portugal OR portuguese republic OR puerto rico OR romania OR russia OR russian federation OR ussr OR soviet union OR union of soviet socialist republics OR rwanda OR ruanda OR samoa OR pacific islands OR polynesia OR samoan islands OR sao tome and principe OR saudi arabia OR senegal OR serbia OR seychelles OR sierra leone OR slovakia OR slovak republic OR slovenia OR melanesia OR solomon island OR solomon islands OR norfolk island OR somalia OR south africa OR south sudan OR sri lanka OR ceylon OR saint kitts and nevis OR st kitts and nevis OR saint lucia OR st lucia OR saint vincent OR st vincent OR grenadines OR sudan OR suriname OR surinam OR syria OR syrian arab republic OR tajikistan OR tadjikistan OR tadzhikistan OR tadzhik OR tanzania OR tanganyika OR thailand OR siam OR timor leste OR east timor OR togo OR togolese republic OR tonga OR trinidad OR tobago OR tunisia OR turkey OR turkmenistan OR turkmen OR uganda OR ukraine OR uruguay OR uzbekistan OR uzbek OR vanuatu OR new hebrides OR venezuela OR vietnam OR viet nam OR middle east OR west bank OR gaza OR palestine OR yemen OR yugoslavia OR zambia OR zimbabwe OR northern rhodesia OR global south OR africa south of the sahara OR sub saharan africa OR subsaharan africa OR central africa OR north africa OR northern africa OR magreb OR maghrib OR sahara OR southern africa OR east africa OR eastern africa OR west africa OR western africa OR west indies OR indian ocean islands OR caribbean OR central america OR latin america OR south america OR central asia OR north asia OR northern asia OR southeastern asia OR south eastern asia OR southeast asia OR south east asia OR western asia OR east europe OR eastern europe OR developing country OR developing countries OR developing nation OR developing nations OR developing population OR developing populations OR developing world OR less developed country OR less developed countries OR less developed nation OR less developed nations OR less developed world OR lesser developed countries OR lesser developed nations OR under developed country OR under developed countries OR under developed nations OR under developed world OR underdeveloped country OR underdeveloped countries OR underdeveloped nation OR underdeveloped nations OR underdeveloped population OR underdeveloped populations OR underdeveloped world OR middle income country OR middle income countries OR middle income nation OR middle	

	income nations OR middle income population OR middle income populations OR low income country OR low income countries OR low income nation OR low income nations OR low income population OR low income populations OR lower income country OR lower income countries OR lower income nations OR lower income population OR lower income populations OR underserved countries OR underserved nations OR underserved population OR underserved populations OR under served population OR under served populations OR deprived countries OR deprived population OR deprived populations OR poor country OR poor countries OR poor nation OR poor nations OR poor population OR poor populations OR poor world OR poorer countries OR poorer nations OR poorer population OR poorer populations OR developing economy OR developing economies OR less developed economy OR less developed economies OR underdeveloped economies OR middle income economy OR middle income economies OR low income economy OR low income economies OR lower income economies OR low gdp OR low gnp OR low gross domestic OR low gross national OR lower gdp OR lower gross domestic OR lmic OR lmics OR third world OR lami country OR lami countries OR transitional country OR transitional countries OR emerging economies OR emerging nation OR emerging nations	
	AND	
**Research type**	Qualitative research OR Qualitative Method* OR Qualitative OR Focus group* OR Interview* OR Mixed Method*	610,278 title/abstract
**TOTAL**	***Filters applied:*** **Review.**	93

Search copied from PubMed; 12/02/22 N=93

((service user OR caregiver OR health personnel[MeSH Major Topic]) OR (service user*[Title/Abstract] OR consumer*[Title/Abstract] **OR**

patient*[Title/Abstract] OR stakeholder*[Title/Abstract] OR user*[Title/Abstract] OR client*[Title/Abstract] OR carer*[Title/Abstract] OR caregiver*[Title/Abstract] OR parent*[Title/Abstract] OR family[Title/Abstract] OR relative*[Title/Abstract] OR guardian*[Title/Abstract] OR Health worker[Title/Abstract] OR Health care worker[Title/Abstract] OR Healthcare worker[Title/Abstract] OR Health Care Provider*[Title/Abstract] OR Healthcare Provider[Title/Abstract] OR Health professional[Title/Abstract] OR Health Care Professional[Title/Abstract] OR Healthcare Professional[Title/Abstract] OR Health staff[Title/Abstract] OR Medical staff[Title/Abstract] OR clinical staff[Title/Abstract] OR medical workforce[Title/Abstract] OR medical work force[Title/Abstract] OR health workforce[Title/Abstract] OR health work force[Title/Abstract]))

AND

((Mental Disorders/prevention and control OR Nervous System Diseases/prevention and control OR Substance-Related Disorders/prevention and control OR Epilepsy/prevention and control OR Mental Health Services[MeSH Major Topic]) **OR**

(Mental Health Service[Title/Abstract] OR Mental Healthcare[Title/Abstract] OR Mental Health Care[Title/Abstract] OR Mental Health System[Title/Abstract] OR Psychiatric Services[Title/Abstract] OR Psychiatric Care[Title/Abstract] OR Psychiatric Health Care[Title/Abstract] OR Psychiatric Healthcare[Title/Abstract] OR Mental Illness[Title/Abstract] OR Mental Health[Title/Abstract] OR Severe Mental Disorder[Title/Abstract] OR Common Mental Disorder[Title/Abstract] OR Alcohol Use Disorder[Title/Abstract] OR Alcohol Abuse[Title/Abstract] OR Alcohol-Related Disorder[Title/Abstract] OR Alcohol Related Disorder[Title/Abstract] OR Alcohol Addiction[Title/Abstract] OR Substance Disorder[Title/Abstract] OR Substance Abuse[Title/Abstract] OR Substance-Use Disorder[Title/Abstract] OR Substance Use Disorder[Title/Abstract] OR Opioid Abuse[Title/Abstract] OR Opiate Addiction[Title/Abstract] OR Opioid-Related Disorder[Title/Abstract] OR Opioid Related Disorder[Title/Abstract] OR Cannabis-Related Disorder[Title/Abstract] OR Cannabis Related Disorder[Title/Abstract] OR Cocaine Related Disorder[Title/Abstract] OR Cocaine Addiction[Title/Abstract] OR Amphetamine-Related Disorder[Title/Abstract] OR Amphetamine Related Disorder[Title/Abstract] OR Amphetamine Addiction[Title/Abstract] OR Heroin Dependence[Title/Abstract] OR Heroin Abuse[Title/Abstract] OR Heroin Addiction[Title/Abstract] OR Substance Induced Psychos[Title/Abstract] OR Substance-Induced Psychos[Title/Abstract] OR Depression[Title/Abstract] OR Depressive Disorder[Title/Abstract] OR Mood Disorder[Title/Abstract] OR Major Depressive Disorder[Title/Abstract] OR Attention Deficit Disorder[Title/Abstract] OR attention deficit hyperactivity disorder[Title/Abstract] OR ADHD[Title/Abstract] OR Conduct Disorder[Title/Abstract] OR Neurocognitive Disorder[Title/Abstract] OR Neurodevelopmental Disorder[Title/Abstract] OR Neurodevelopmental disabilit*[Title/Abstract] OR Developmental Disabilit*[Title/Abstract] OR Developmental Disorder[Title/Abstract] OR Autism Spectrum Disorder[Title/Abstract] OR Autistic Spectrum Disorder[Title/Abstract] OR Aspergers Disease[Title/Abstract] OR Aspergers Syndrome[Title/Abstract] OR learning disabilit*[Title/Abstract] OR intellectual disabilit*[Title/Abstract] OR intellectual development[Title/Abstract] OR mental retardation[Title/Abstract] OR hyperkinetic disorder[Title/Abstract] OR tic disorder[Title/Abstract] OR self-harm*[Title/Abstract] OR self-injur*[Title/Abstract] OR suicid*[Title/Abstract] OR Dementia[Title/Abstract] OR Alzheimer*[Title/Abstract] OR Parkinson*[Title/Abstract] OR Epilep*[Title/Abstract] OR seizure[Title/Abstract] OR Schizophrenia[Title/Abstract] OR Psychosis[Title/Abstract] OR Psychoses[Title/Abstract] OR Psychotic Disorder[Title/Abstract] OR Schizoaffective Disorder[Title/Abstract] OR Schizophreniform Disorder[Title/Abstract] OR Psychotic Affective Disorder[Title/Abstract] OR Psychotic Mood Disorder[Title/Abstract] OR Affective Psychosis[Title/Abstract] OR schizotypal[Title/Abstract] OR delusional[Title/Abstract] OR Bipolar Disorder[Title/Abstract] OR Manic Depressive Psychosis[Title/Abstract] OR Bipolar Affective Psychosis[Title/Abstract] OR Bipolar Affective Disorder[Title/Abstract] OR Manic Depressive Psychoses[Title/Abstract] OR Bipolar Depression[Title/Abstract] OR Post-Traumatic Stress Disorder[Title/Abstract] OR Post Traumatic Stress Disorder[Title/Abstract] OR Traumatic Stress Disorder[Title/Abstract] OR Stress Disorder[Title/Abstract] OR acute stress reaction[Title/Abstract] OR grief[Title/Abstract] OR Anxiety Disorder[Title/Abstract] OR phobi*[Title/Abstract] OR agoraphobi*[Title/Abstract] OR panic disorder[Title/Abstract] OR GAD[Title/Abstract] OR obsessive compulsive disorder[Title/Abstract] OR OCD[Title/Abstract]))

AND

((Patient Acceptance of Health Care OR Delivery of Health Care OR Health Services Accessibility OR Patient Reported Outcome Measures OR Quality of Health Care OR Patient Compliance OR Healthcare Disparities OR Health Services[MeSH Major Topic]) **OR**

(facilitat*[Title/Abstract] OR enhanc*[Title/Abstract] OR enable*[Title/Abstract] OR opportunity*[Title/Abstract] OR encourag*[Title/Abstract] OR motivat*[Title/Abstract] OR promot*[Title/Abstract] OR influen*[Title/Abstract] OR barrier*[Title/Abstract] OR challenge*[Title/Abstract] OR block*[Title/Abstract] OR challeng*[Title/Abstract] OR constrain*[Title/Abstract] OR deter*[Title/Abstract] OR difficult*[Title/Abstract] OR discourag*[Title/Abstract] OR disincentive*[Title/Abstract] OR encumber*[Title/Abstract] OR encumbranc*[Title/Abstract] OR hinder*[Title/Abstract] OR hindrance*[Title/Abstract] OR impair*[Title/Abstract] OR impede*[Title/Abstract] OR impeding[Title/Abstract] OR impediment*[Title/Abstract] OR limit*[Title/Abstract] OR delay[Title/Abstract] OR obstruct*[Title/Abstract] OR problem[Title/Abstract] OR restrain*[Title/Abstract] OR restrict*[Title/Abstract] OR interfer*[Title/Abstract] OR perceive*[Title/Abstract] OR perception*[Title/Abstract] OR perspective*[Title/Abstract] OR view*[Title/Abstract] OR experience*[Title/Abstract] OR need*[Title/Abstract] OR attitude*[Title/Abstract] OR belie*[Title/Abstract] OR opinion*[Title/Abstract] OR quality[Title/Abstract] OR Implementat*[Title/Abstract] OR adoption[Title/Abstract] OR patient experience[Title/Abstract] OR uptake*[Title/Abstract] OR utilis*[Title/Abstract] OR utiliz*[Title/Abstract] OR ((use[Title/Abstract] OR acceptance[Title/Abstract] OR acceptability[Title/Abstract] OR availability[Title/Abstract] OR accessibility[Title/Abstract] OR access[Title/Abstract] OR accessing[Title/Abstract] OR receipt[Title/Abstract] OR receive[Title/Abstract] OR received[Title/Abstract] OR receiving[Title/Abstract]) AND (health care[Title/Abstract] OR healthcare[Title/Abstract] OR patient care[Title/Abstract] OR health service*[Title/Abstract] OR primary care[Title/Abstract] OR visit[Title/Abstract] OR appointment[Title/Abstract])) OR ((provide[Title/Abstract] OR providing[Title/Abstract] OR provis*[Title/Abstract] OR distribut*[Title/Abstract] OR deliver*[Title/Abstract]) AND OR[Title/Abstract] OR OR[Title/Abstract] OR OR[Title/Abstract]))

AND

(afghanistan[Title/Abstract] OR albania[Title/Abstract] OR algeria[Title/Abstract] OR american samoa[Title/Abstract] OR angola[Title/Abstract] OR antigua[Title/Abstract] OR barbuda[Title/Abstract] OR argentina[Title/Abstract] OR armenia[Title/Abstract] OR armenian[Title/Abstract] OR aruba[Title/Abstract] OR azerbaijan[Title/Abstract] OR bahrain[Title/Abstract] OR bangladesh[Title/Abstract] OR barbados[Title/Abstract] OR belarus[Title/Abstract] OR byelarus[Title/Abstract] OR belorussia[Title/Abstract] OR byelorussian[Title/Abstract] OR belize[Title/Abstract] OR british honduras[Title/Abstract] OR benin[Title/Abstract] OR dahomey[Title/Abstract] OR bhutan[Title/Abstract] OR bolivia[Title/Abstract] OR bosnia[Title/Abstract] OR herzegovina[Title/Abstract] OR botswana[Title/Abstract] OR bechuanaland[Title/Abstract] OR brazil[Title/Abstract] OR brasil[Title/Abstract] OR bulgaria[Title/Abstract] OR burkina faso[Title/Abstract] OR burkina fasso[Title/Abstract] OR upper volta[Title/Abstract] OR burundi[Title/Abstract] OR urundi[Title/Abstract] OR cabo verde[Title/Abstract] OR cape verde[Title/Abstract] OR cambodia[Title/Abstract] OR kampuchea[Title/Abstract] OR khmer republic[Title/Abstract] OR cameroon[Title/Abstract] OR cameron[Title/Abstract] OR cameroun[Title/Abstract] OR central african republic[Title/Abstract] OR ubangi shari[Title/Abstract] OR chad[Title/Abstract] OR chile[Title/Abstract] OR china[Title/Abstract] OR

colombia[Title/Abstract] OR comoros[Title/Abstract] OR comoro islands[Title/Abstract] OR mayotte[Title/Abstract] OR congo[Title/Abstract] OR zaire[Title/Abstract] OR costa rica[Title/Abstract] OR cote d'ivoire[Title/Abstract] OR cote d' ivoire[Title/Abstract] OR cote divoire[Title/Abstract] OR cote d ivoire[Title/Abstract] OR ivory coast[Title/Abstract] OR croatia[Title/Abstract] OR cuba[Title/Abstract] OR cyprus[Title/Abstract] OR czech republic[Title/Abstract] OR czechoslovakia[Title/Abstract] OR djibouti[Title/Abstract] OR french somaliland[Title/Abstract] OR dominica[Title/Abstract] OR dominican republic[Title/Abstract] OR ecuador[Title/Abstract] OR egypt[Title/Abstract] OR united arab republic[Title/Abstract] OR el salvador[Title/Abstract] OR equatorial guinea[Title/Abstract] OR spanish guinea[Title/Abstract] OR eritrea[Title/Abstract] OR estonia[Title/Abstract] OR eswatini[Title/Abstract] OR swaziland[Title/Abstract] OR ethiopia[Title/Abstract] OR fiji[Title/Abstract] OR gabon[Title/Abstract] OR gabonese republic[Title/Abstract] OR gambia[Title/Abstract] OR georgia[Title/Abstract] OR georgian[Title/Abstract] OR ghana[Title/Abstract] OR gold coast[Title/Abstract] OR gibraltar[Title/Abstract] OR greece[Title/Abstract] OR grenada[Title/Abstract] OR guam[Title/Abstract] OR guatemala[Title/Abstract] OR guinea[Title/Abstract] OR guyana[Title/Abstract] OR guiana[Title/Abstract] OR haiti[Title/Abstract] OR hispaniola[Title/Abstract] OR honduras[Title/Abstract] OR hungary[Title/Abstract] OR india[Title/Abstract] OR indonesia[Title/Abstract] OR timor[Title/Abstract] OR iran[Title/Abstract] OR iraq[Title/Abstract] OR isle of man[Title/Abstract] OR jamaica[Title/Abstract] OR jordan[Title/Abstract] OR kazakhstan[Title/Abstract] OR kazakh[Title/Abstract] OR kenya[Title/Abstract] OR korea[Title/Abstract] OR kosovo[Title/Abstract] OR kyrgyzstan[Title/Abstract] OR kirghizia[Title/Abstract] OR kirgizstan[Title/Abstract] OR kyrgyz republic[Title/Abstract] OR kirghiz[Title/Abstract] OR laos[Title/Abstract] OR lao pdr[Title/Abstract] OR lao people's democratic republic[Title/Abstract] OR latvia[Title/Abstract] OR lebanon[Title/Abstract] OR lesotho[Title/Abstract] OR basutoland[Title/Abstract] OR liberia[Title/Abstract] OR libya[Title/Abstract] OR libyan arab jamahiriya[Title/Abstract] OR lithuania[Title/Abstract] OR macau[Title/Abstract] OR macao[Title/Abstract] OR macedonia[Title/Abstract] OR madagascar[Title/Abstract] OR malagasy republic[Title/Abstract] OR malawi[Title/Abstract] OR nyasaland[Title/Abstract] OR malaysia[Title/Abstract] OR maldives[Title/Abstract] OR indian ocean[Title/Abstract] OR mali[Title/Abstract] OR malta[Title/Abstract] OR micronesia[Title/Abstract] OR kiribati[Title/Abstract] OR marshall islands[Title/Abstract] OR nauru[Title/Abstract] OR northern mariana islands[Title/Abstract] OR palau[Title/Abstract] OR tuvalu[Title/Abstract] OR mauritania[Title/Abstract] OR mauritius[Title/Abstract] OR mexico[Title/Abstract] OR moldova[Title/Abstract] OR moldovian[Title/Abstract] OR mongolia[Title/Abstract] OR montenegro[Title/Abstract] OR morocco[Title/Abstract] OR ifni[Title/Abstract] OR mozambique[Title/Abstract] OR portuguese east africa[Title/Abstract] OR myanmar[Title/Abstract] OR burma[Title/Abstract] OR namibia[Title/Abstract] OR nepal[Title/Abstract] OR netherlands antilles[Title/Abstract] OR nicaragua[Title/Abstract] OR niger[Title/Abstract] OR nigeria[Title/Abstract] OR oman[Title/Abstract] OR muscat[Title/Abstract] OR pakistan[Title/Abstract] OR panama[Title/Abstract] OR papua new guinea[Title/Abstract] OR paraguay[Title/Abstract] OR peru[Title/Abstract] OR philippines[Title/Abstract] OR philipines[Title/Abstract] OR phillipines[Title/Abstract] OR phillippines[Title/Abstract] OR poland[Title/Abstract] OR polish people's republic[Title/Abstract] OR portugal[Title/Abstract] OR portuguese republic[Title/Abstract] OR puerto rico[Title/Abstract] OR romania[Title/Abstract] OR russia[Title/Abstract] OR russian federation[Title/Abstract] OR ussr[Title/Abstract] OR soviet union[Title/Abstract] OR union of soviet socialist republics[Title/Abstract] OR rwanda[Title/Abstract] OR ruanda[Title/Abstract] OR samoa[Title/Abstract] OR pacific islands[Title/Abstract] OR polynesia[Title/Abstract] OR samoan islands[Title/Abstract] OR sao tome[Title/Abstract] AND principe[Title/Abstract] OR saudi arabia[Title/Abstract] OR senegal[Title/Abstract] OR serbia[Title/Abstract] OR seychelles[Title/Abstract] OR sierra leone[Title/Abstract] OR slovakia[Title/Abstract] OR slovak

republic[Title/Abstract] OR slovenia[Title/Abstract] OR melanesia[Title/Abstract] OR solomon island[Title/Abstract] OR solomon islands[Title/Abstract] OR norfolk island[Title/Abstract] OR somalia[Title/Abstract] OR south africa[Title/Abstract] OR south sudan[Title/Abstract] OR sri lanka[Title/Abstract] OR ceylon[Title/Abstract] OR saint kitts[Title/Abstract] AND nevis[Title/Abstract] OR st kitts[Title/Abstract] AND saint lucia[Title/Abstract] OR st lucia[Title/Abstract] OR saint vincent[Title/Abstract] OR st vincent[Title/Abstract] OR grenadines[Title/Abstract] OR sudan[Title/Abstract] OR suriname[Title/Abstract] OR surinam[Title/Abstract] OR syria[Title/Abstract] OR syrian arab republic[Title/Abstract] OR tajikistan[Title/Abstract] OR tadjikistan[Title/Abstract] OR tadzhikistan[Title/Abstract] OR tadzhik[Title/Abstract] OR tanzania[Title/Abstract] OR tanganyika[Title/Abstract] OR thailand[Title/Abstract] OR siam[Title/Abstract] OR timor leste[Title/Abstract] OR east timor[Title/Abstract] OR togo[Title/Abstract] OR togolese republic[Title/Abstract] OR tonga[Title/Abstract] OR trinidad[Title/Abstract] OR tobago[Title/Abstract] OR tunisia[Title/Abstract] OR turkey[Title/Abstract] OR turkmenistan[Title/Abstract] OR turkmen[Title/Abstract] OR uganda[Title/Abstract] OR ukraine[Title/Abstract] OR uruguay[Title/Abstract] OR uzbekistan[Title/Abstract] OR uzbek[Title/Abstract] OR vanuatu[Title/Abstract] OR new hebrides[Title/Abstract] OR venezuela[Title/Abstract] OR vietnam[Title/Abstract] OR viet nam[Title/Abstract] OR middle east[Title/Abstract] OR west bank[Title/Abstract] OR gaza[Title/Abstract] OR palestine[Title/Abstract] OR yemen[Title/Abstract] OR yugoslavia[Title/Abstract] OR zambia[Title/Abstract] OR zimbabwe[Title/Abstract] OR northern rhodesia[Title/Abstract] OR global south[Title/Abstract] OR africa south of the sahara[Title/Abstract] OR sub saharan africa[Title/Abstract] OR subsaharan africa[Title/Abstract] OR central africa[Title/Abstract] OR north africa[Title/Abstract] OR northern africa[Title/Abstract] OR magreb[Title/Abstract] OR maghrib[Title/Abstract] OR sahara[Title/Abstract] OR southern africa[Title/Abstract] OR east africa[Title/Abstract] OR eastern africa[Title/Abstract] OR west africa[Title/Abstract] OR western africa[Title/Abstract] OR west indies[Title/Abstract] OR indian ocean islands[Title/Abstract] OR caribbean[Title/Abstract] OR central america[Title/Abstract] OR latin america[Title/Abstract] OR south america[Title/Abstract] OR central asia[Title/Abstract] OR north asia[Title/Abstract] OR northern asia[Title/Abstract] OR southeastern asia[Title/Abstract] OR south eastern asia[Title/Abstract] OR southeast asia[Title/Abstract] OR south east asia[Title/Abstract] OR western asia[Title/Abstract] OR east europe[Title/Abstract] OR eastern europe[Title/Abstract] OR developing country[Title/Abstract] OR developing countries[Title/Abstract] OR developing nation[Title/Abstract] OR developing nations[Title/Abstract] OR developing population[Title/Abstract] OR developing populations[Title/Abstract] OR developing world[Title/Abstract] OR less developed country[Title/Abstract] OR less developed countries[Title/Abstract] OR less developed nation[Title/Abstract] OR less developed nations[Title/Abstract] OR less developed world[Title/Abstract] OR lesser developed countries[Title/Abstract] OR lesser developed nations[Title/Abstract] OR under developed country[Title/Abstract] OR under developed countries[Title/Abstract] OR under developed nations[Title/Abstract] OR under developed world[Title/Abstract] OR underdeveloped country[Title/Abstract] OR underdeveloped countries[Title/Abstract] OR underdeveloped nation[Title/Abstract] OR underdeveloped nations[Title/Abstract] OR underdeveloped population[Title/Abstract] OR underdeveloped populations[Title/Abstract] OR underdeveloped world[Title/Abstract] OR middle income country[Title/Abstract] OR middle income countries[Title/Abstract] OR middle income nation[Title/Abstract] OR middle income nations[Title/Abstract] OR middle income population[Title/Abstract] OR middle income populations[Title/Abstract] OR low income country[Title/Abstract] OR low income countries[Title/Abstract] OR low income nation[Title/Abstract] OR low income nations[Title/Abstract] OR low income population[Title/Abstract] OR low income populations[Title/Abstract] OR lower income country[Title/Abstract] OR lower income countries[Title/Abstract] OR lower income nations[Title/Abstract] OR lower income population[Title/Abstract] OR lower income

populations[Title/Abstract] OR underserved countries[Title/Abstract] OR underserved nations[Title/Abstract] OR underserved population[Title/Abstract] OR underserved populations[Title/Abstract] OR under served population[Title/Abstract] OR under served populations[Title/Abstract] OR deprived countries[Title/Abstract] OR deprived population[Title/Abstract] OR deprived populations[Title/Abstract] OR poor country[Title/Abstract] OR poor countries[Title/Abstract] OR poor nation[Title/Abstract] OR poor nations[Title/Abstract] OR poor population[Title/Abstract] OR poor populations[Title/Abstract] OR poor world[Title/Abstract] OR poorer countries[Title/Abstract] OR poorer nations[Title/Abstract] OR poorer population[Title/Abstract] OR poorer populations[Title/Abstract] OR developing economy[Title/Abstract] OR developing economies[Title/Abstract] OR less developed economy[Title/Abstract] OR less developed economies[Title/Abstract] OR underdeveloped economies[Title/Abstract] OR middle income economy[Title/Abstract] OR middle income economies[Title/Abstract] OR low income economy[Title/Abstract] OR low income economies[Title/Abstract] OR lower income economies[Title/Abstract] OR low gdp[Title/Abstract] OR low gnp[Title/Abstract] OR low gross domestic[Title/Abstract] OR low gross national[Title/Abstract] OR lower gdp[Title/Abstract] OR lower gross domestic[Title/Abstract] OR lmic[Title/Abstract] OR lmics[Title/Abstract] OR third world[Title/Abstract] OR lami country[Title/Abstract] OR lami countries[Title/Abstract] OR transitional country[Title/Abstract] OR transitional countries[Title/Abstract] OR emerging economies[Title/Abstract] OR emerging nation[Title/Abstract] OR emerging nations[Title/Abstract]))

AND

(Qualitative research[Title/Abstract] OR Qualitative Method*[Title/Abstract] OR Qualitative[Title/Abstract] OR Focus group*[Title/Abstract] OR Interview*[Title/Abstract] OR Mixed Method*[Title/Abstract])

AND (review[Filter])

Sources used to develop search terms

**Population**:	Service users<br>https://www.crd.york.ac.uk/prospero/display_record.php?RecordID=150940 Review: "What are the barriers and enablers to clinical implementation of multi-drug pharmacogenetic (PGx) testing?"<br>https://www.crd.york.ac.uk/prospero/display_record.php?RecordID=116712 >>> protocol strategy: https://www.crd.york.ac.uk/PROSPEROFILES/116712_STRATEGY_20181120.pdf Protocol Bjorkqvist et al "An evaluation

	of existing models of patient and public involvement in the development of clinical practice guidelines: a systematic review"<br><br>caregivers<br><br>https://www.crd.york.ac.uk/prospero/display_record.php?RecordID=288437 >>> protocol strategy: https://www.crd.york.ac.uk/PROSPEROFILES/288437_STRATEGY_20211208.pdf A systematic review of participatory research with carers: the methods and outcomes of working with carers to conduct health research<br><br>https://www.crd.york.ac.uk/prospero/display_record.php?RecordID=52509 >>> protocol strategy: https://prism.ucalgary.ca/bitstream/handle/1880/51752/ALL%20SEARCHES.pdf?sequence=1&isAllowed=y A systematic review of caregiver-mediated interventions in patient care settings<br><br>care providers<br><br>https://www.crd.york.ac.uk/prospero/display_record.php?RecordID=46154 https://www.crd.york.ac.uk/PROSPEROFILES/46154_STRATEGY_20160718.pdf Rowe et al., " A systematic review of the effectiveness and costs of interventions to improve health care provider performance and related health outcomes in low- and middle-income countries"; protocol strategy:<br><br>https://www.crd.york.ac.uk/prospero/display_record.php?RecordID=215507 >>> protocol strategy: https://www.crd.york.ac.uk/PROSPEROFILES/215507_STRATEGY_20201020.pdf Assessment of COVID-19 effect on healthcare workers' mental health, systematic review
**Intervention**	https://pubmed.ncbi.nlm.nih.gov/24119375/ Mutamba et al., 2013: " Roles and effectiveness of lay community health workers in the **prevention of mental, neurological and substance use disorders in** low and middle income countries: a systematic review"<br><br>https://www.ncbi.nlm.nih.gov/pmc/articles/PMC7181535/ >>> protocol strategy file:///C:/Users/Petra/Downloads/13561_2020_268_MOESM1_ESM.pdf Docrat 2020 "The impact of social, national and community-based health insurance **on health care utilization for mental, neurological and substance-use disorders** in low- and middle-income countries: a systematic review"; PROTOCOL

	https://www.crd.york.ac.uk/prospero/display_record.php?RecordID=299682 >> search strategy https://www.crd.york.ac.uk/PROSPEROFILES/299682_STRATEGY_20211221.pdf Heim et al., " Research evidence on how to reduce stigma and discrimination related to **mental and substance use disorders**: umbrella review"<br><br>Terms targeted to priority conditions addressed by mhGAP (https://www.who.int/activities/scaling-up-mental-health-care), and conditions in PICOs for this mhGAP update, as per annex shared by Elaine.
**Context**	Cochrane 2020 filter; https://epoc.cochrane.org/lmic-filters >> https://epoc.cochrane.org/sites/epoc.cochrane.org/files/public/uploads/epoc_lmic_filters_2020_v4.docx<br><br>Filter includes terms as Text Words, I ran search with both text words and title/abstract search. Latter more feasible.
**Outcome**	https://www.crd.york.ac.uk/prospero/display_record.php?RecordID=150940 Review: "What are the barriers and enablers to clinical implementation of multi-drug pharmacogenetic (PGx) testing?"<br><br>https://www.crd.york.ac.uk/prospero/display_record.php?RecordID=214528 >>> https://www.crd.york.ac.uk/PROSPEROFILES/214528_STRATEGY_20201015.pdf Protocol, Thomas et al., "A systematic review of qualitative studies investigating service-user experiences of constant observations, whilst admitted to an inpatient psychiatric hospital"<br><br>https://bmchealthservres.biomedcentral.com/articles/10.1186/1472-6963-14-2#MOESM1 >> search strategy here: https://static-content.springer.com/esm/art%3A10.1186%2F1472-6963-14-2/MediaObjects/12913_2013_3733_MOESM1_ESM.docx Oliver et al, 2014 "A systematic review of barriers to and facilitators of the use of evidence by policymakers"<br><br>https://doi.org/10.1186/s13012-016-0370-1 >> search strategy here: https://static-content.springer.com/esm/art%3A10.1186%2Fs13012-016-0370-1/MediaObjects/13012_2016_370_MOESM1_ESM.docx Tricco, A.C., Cardoso, R., Thomas, S.M. *et al.* Barriers and facilitators to uptake of systematic reviews by policy makers and health care managers: a scoping review. *Implementation Sci* **11,** 4 (2015).<br><br>https://www.crd.york.ac.uk/prospero/display_record.php?RecordID=133990 >> protocol search strategy https://www.crd.york.ac.uk/PROSPEROFILES/133990_STRATEGY_20190430.pdf Wouk et al, A systematic review of health system-, provider-, and patient-level predictors of postpartum healthcare utilization by low-income, uninsured, and/or minority mothers in the United States

	https://www.crd.york.ac.uk/prospero/display_record.php?RecordID=148700 >>> protocol strategy https://www.crd.york.ac.uk/PROSPEROFILES/148700_STRATEGY_20191106.pdf A qualitative evidence synthesis of barriers and facilitators to assisted dying: A focus on health professionals' perspectives
Research type	https://www.crd.york.ac.uk/prospero/display_record.php?RecordID=148700 >>> protocol strategy https://www.crd.york.ac.uk/PROSPEROFILES/148700_STRATEGY_20191106.pdf A qualitative evidence synthesis of barriers and facilitators to assisted dying: A focus on health professionals' perspectives.
Design	Used PubMed filter; tested both “review” and “systematic review”

EMBASE

**Domain**	**Search terms (MeSH term and/or Key words)**
**Population**	health care personnel/ OR caregiver/
	OR
	(service user* or consumer* or patient* or stakeholder* or user* or client* or carer* or caregiver* or parent* or family or relative* or guardian* or Health worker or Health care worker or Healthcare worker or Health Care Provider* or Healthcare Provider or Health professional or Health Care Professional or Healthcare Professional or Health staff or Medical staff or clinical staff or medical workforce or medical work force or health workforce or health work force).ti,ab.
	AND
**Intervention**	Drug dependence treatment/ OR Mental Health Services/
	OR

Care for mental, neurological and substance use disorders	(Mental Health Service or Mental Healthcare or Mental Health Care or Mental Health System or Psychiatric Services or Psychiatric Care or Psychiatric Health Care or Psychiatric Healthcare or Mental Illness or Mental Health or Severe Mental Disorder or Common Mental Disorder or "Alcohol Use Disorder" or Alcohol Related Disorder or Alcohol Addiction or Substance Disorder or Substance Abuse or "Substance-Use Disorder" or "Substance Use Disorder" or Opioid Abuse or Opiate Addiction or Opioid-Related Disorder or Opioid Related Disorder or "Cannabis-Related Disorder" or Cannabis Related Disorder or Cocaine Related Disorder or Cocaine Related Disorder or Cocaine Addiction or "Amphetamine-Related Disorder" or Amphetamine Related Disorder or Amphetamine Addiction or Heroin Dependence or Heroin Abuse or Heroin Addiction or Substance Induced Psychos or "Substance-Induced Psychos" or Depression or Depressive Disorder or Mood Disorder or Major Depressive Disorder or Attention Deficit Disorder or attention deficit hyperactivity disorder or ADHD or Conduct Disorder or Neurocognitive Disorder or Neurodevelopmental Disorder or "Neurodevelopmental disabilit*" or Developmental Disabilit* or Developmental Disorder or Autism Spectrum Disorder or Autistic Spectrum Disorder or Aspergers Disease or Aspergers Syndrome or learning disabilit* or intellectual disabilit* or intellectual development or mental retardation or hyperkinetic disorder or tic disorder or "self-harm*" or "self-injur*" or suicid* or Dementia or Alzheimer* or Parkinson* or Epilep* or seizure or Schizophrenia or Psychosis or Psychoses or Psychotic Disorder or Schizoaffective Disorder or Schizophreniform Disorder or Psychotic Affective Disorder or Psychotic Mood Disorder or Affective Psychosis or schizotypal or delusional or Bipolar Disorder or Manic Depressive Psychosis or Bipolar Affective Psychosis or Bipolar Affective Disorder or Manic Depressive Psychoses or Bipolar Depression or "Post-Traumatic Stress Disorder" or Post Traumatic Stress Disorder or Traumatic Stress Disorder or Stress Disorder or acute stress reaction or grief or Anxiety Disorder or phobi* or agoraphobi* or panic disorder or GAD or obsessive compulsive disorder or OCD).ti,ab.
	AND
**Outcome**<br>views and experience of care *uptake* and/or care *provision;* factors (barriers/facilitators) influencing service *uptake* and/or care *provision*	health care delivery/ or health care access/ or patient-reported outcome/ or health care quality/ or patient compliance/. or health care disparity/ or health service/ or patient attitude/
	OR
	(facilitat* or enhanc* or enable* or opportunity* or encourag* or motivat* or promot* or influen* or barrier* or challenge* or block* or challeng* or constrain* or deter* or difficult* or discourag* or disincentive* or encumber* or encumbranc* or hinder* or hindrance* or impair* or impede* or impeding or impediment* or limit* or delay or obstruct* or problem or restrain* or restrict* or interfer* or perceive* or perception* or perspective* or view* or experience* or need* or attitude* or belie* or opinion* or quality or Implementat* or adoption or patient experience or uptake* or utilis* or utiliz* or (("use" or acceptance or acceptability or availability or accessibility or access or accessing or receipt or receive or received or receiving) and (health care or healthcare or patient care or

	health service* or primary care or visit or appointment)) or ((provide or providing or provis* or distribut* or deliver*) and (health care or healthcare or patient care or health service* or primary care or visit or appointment))).ti,ab.
	AND
**Context**<br>*LMICs*	(afghanistan OR albania OR algeria OR american samoa OR angola OR "antigua and barbuda" OR antigua OR barbuda OR argentina OR armenia OR armenian OR aruba OR azerbaijan OR bahrain OR bangladesh OR barbados OR republic of belarus OR belarus OR byelarus OR belorussia OR byelorussian OR belize OR british honduras OR benin OR dahomey OR bhutan OR bolivia OR "bosnia and herzegovina" OR bosnia OR herzegovina OR botswana OR bechuanaland OR brazil OR brasil OR bulgaria OR burkina faso OR burkina fasso OR upper volta OR burundi OR urundi OR cabo verde OR cape verde OR cambodia OR kampuchea OR khmer republic OR cameroon OR cameron OR cameroun OR central african republic OR ubangi shari OR chad OR chile OR china OR colombia OR comoros OR comoro islands OR iles comores OR mayotte OR democratic republic of the congo OR democratic republic congo OR congo OR zaire OR costa rica OR "cote d'ivoire" OR "cote d' ivoire" OR cote divoire OR cote d ivoire OR ivory coast OR croatia OR cuba OR cyprus OR czech republic OR czechoslovakia OR djibouti OR french somaliland OR dominica OR dominican republic OR ecuador OR egypt OR united arab republic OR el salvador OR equatorial guinea OR spanish guinea OR eritrea OR estonia OR eswatini OR swaziland OR ethiopia OR fiji OR gabon OR gabonese republic OR gambia OR "georgia (republic)" OR georgian OR ghana OR gold coast OR gibraltar OR greece OR grenada OR guam OR guatemala OR guinea OR guinea bissau OR guyana OR british guiana OR haiti OR hispaniola OR honduras OR hungary OR india OR indonesia OR timor OR iran OR iraq OR isle of man OR jamaica OR jordan OR kazakhstan OR kazakh OR kenya OR "democratic people's republic of korea" OR republic of korea OR north korea OR south korea OR korea OR kosovo OR kyrgyzstan OR kirghizia OR kirgizstan OR kyrgyz republic OR kirghiz OR laos OR lao pdr OR "lao people's democratic republic" OR latvia OR lebanon OR lebanese republic OR lesotho OR basutoland OR liberia OR libya OR libyan arab jamahiriya OR lithuania OR macau OR macao OR republic of north macedonia OR macedonia OR madagascar OR malagasy republic OR malawi OR nyasaland OR malaysia OR malay federation OR malaya federation OR maldives OR indian ocean islands OR indian ocean OR mali OR malta OR micronesia OR federated states of micronesia OR kiribati OR marshall islands OR nauru OR northern mariana islands OR palau OR tuvalu OR mauritania OR mauritius OR mexico OR moldova OR moldovian OR mongolia OR montenegro OR "montenegro (republic)" OR morocco OR ifni OR mozambique OR portuguese east africa OR myanmar OR burma OR namibia OR nepal OR netherlands antilles OR nicaragua OR niger OR nigeria OR oman OR muscat OR pakistan OR panama OR papua new guinea OR new guinea OR paraguay OR peru OR philippines OR philipines OR phillipines OR phillippines OR poland OR "polish people's republic" OR portugal OR portuguese republic OR puerto rico OR romania OR russia OR russian federation OR ussr OR soviet union OR union of soviet socialist republics OR rwanda OR ruanda OR samoa OR pacific islands OR polynesia OR samoan islands OR navigator island OR navigator islands OR "sao tome and principe" OR saudi arabia OR senegal OR serbia OR seychelles OR sierra leone OR slovakia OR slovak republic OR slovenia OR

	melanesia OR solomon island OR solomon islands OR norfolk island OR norfolk islands OR somalia OR south africa OR south sudan OR sri lanka OR ceylon OR "saint kitts and nevis" OR "st. kitts and nevis" OR saint lucia OR "st. lucia" OR "saint vincent and the grenadines" OR saint vincent OR "st. vincent" OR grenadines OR sudan OR suriname OR surinam OR dutch guiana OR netherlands guiana OR syria OR syrian arab republic OR tajikistan OR tadjikistan OR tadzhikistan OR tadzhik OR tanzania OR tanganyika OR thailand OR siam OR timor leste OR east timor OR togo OR togolese republic OR tonga OR "trinidad and tobago" OR trinidad OR tobago OR tunisia OR "turkey (republic)" OR turkey OR turkmenistan OR turkmen OR uganda OR ukraine OR uruguay OR uzbekistan OR uzbek OR vanuatu OR new hebrides OR venezuela OR vietnam OR viet nam OR middle east OR west bank OR gaza OR palestine OR yemen OR yugoslavia OR zambia OR zimbabwe OR northern rhodesia OR global south OR africa south of the sahara OR "sub saharan africa" OR subsaharan africa OR africa, central OR central africa OR africa, northern OR north africa OR northern africa OR magreb OR maghrib OR sahara OR africa, southern OR southern africa OR africa, eastern OR east africa OR eastern africa OR africa, western OR west africa OR western africa OR west indies OR indian ocean islands OR caribbean region OR caribbean islands OR caribbean OR central america OR latin america OR "south and central america" OR south america OR asia, central OR central asia OR asia, northern OR north asia OR northern asia OR asia, southeastern OR southeastern asia OR south eastern asia OR southeast asia OR south east asia OR asia, western OR western asia OR europe, eastern OR east europe OR eastern europe OR developing country OR developing countries OR developing nation? OR developing population? OR developing world OR less developed countr* OR less developed nation? OR less developed population? OR less developed world OR lesser developed countr* OR lesser developed nation? OR lesser developed population? OR lesser developed world OR under developed countr* OR under developed nation? OR under developed population? OR under developed world OR underdeveloped countr* OR underdeveloped nation? OR underdeveloped population? OR underdeveloped world OR middle income countr* OR middle income nation? OR middle income population? OR low income countr* OR low income nation? OR low income population? OR lower income countr* OR lower income nation? OR lower income population? OR underserved countr* OR underserved nation? OR underserved population? OR underserved world OR under served countr* OR under served nation? OR under served population? OR under served world OR deprived countr* OR deprived nation? OR deprived population? OR deprived world OR poor countr* OR poor nation? OR poor population? OR poor world OR poorer countr* OR poorer nation? OR poorer population? OR poorer world OR developing econom* OR less developed econom* OR lesser developed econom* OR under developed econom* OR underdeveloped econom* OR middle income econom* OR low income econom* OR lower income econom* OR low gdp OR low gnp OR low gross domestic OR low gross national OR lower gdp OR lower gnp OR lower gross domestic OR lower gross national OR lmic OR lmics OR third world OR lami countr* OR transitional countr* OR emerging economies OR emerging nation?).ti,ab
	AND

**Research type**	Qualitative research OR Qualitative Method* OR Qualitative OR Focus group* OR Interview* OR Mixed Method*

SEARCH STRATEGY RETRIEVED FROM EMBASE on 07.03.22

1. health care personnel/ or caregiver/
2. (service user* or consumer* or patient* or stakeholder* or user* or client* or carer* or caregiver* or parent* or family or relative* or guardian* or Health worker or Health care worker or Healthcare worker or Health Care Provider* or Healthcare Provider or Health professional or Health Care Professional or Healthcare Professional or Health staff or Medical staff or clinical staff or medical workforce or medical work force or health workforce or health work force).ti,ab.
3. (Mental Health Service or Mental Healthcare or Mental Health Care or Mental Health System or Psychiatric Services or Psychiatric Care or Psychiatric Health Care or Psychiatric Healthcare or Mental Illness or Mental Health or Severe Mental Disorder or Common Mental Disorder or "Alcohol Use Disorder" or Alcohol Related Disorder or Alcohol Addiction or Substance Disorder or Substance Abuse or "Substance-Use Disorder" or "Substance Use Disorder" or Opioid Abuse or Opiate Addiction or Opioid-Related Disorder or Opioid Related Disorder or "Cannabis-Related Disorder" or Cannabis Related Disorder or Cocaine Related Disorder or Cocaine Related Disorder or Cocaine Addiction or "Amphetamine-Related Disorder" or Amphetamine Related Disorder or Amphetamine Addiction or Heroin Dependence or Heroin Abuse or Heroin Addiction or Substance Induced Psychos or "Substance-Induced Psychos" or Depression or Depressive Disorder or Mood Disorder or Major Depressive Disorder or Attention Deficit Disorder or attention deficit hyperactivity disorder or ADHD or Conduct Disorder or Neurocognitive Disorder or Neurodevelopmental Disorder or "Neurodevelopmental disabilit*" or Developmental Disabilit* or Developmental Disorder or Autism Spectrum Disorder or Autistic Spectrum Disorder or Aspergers Disease or Aspergers Syndrome or learning disabilit* or intellectual disabilit* or intellectual development or mental retardation or hyperkinetic disorder or tic disorder or "self-harm*" or "self-injur*" or suicid* or Dementia or Alzheimer* or Parkinson* or Epilep* or seizure or Schizophrenia or Psychosis or Psychoses or Psychotic Disorder or Schizoaffective Disorder or Schizophreniform Disorder or Psychotic Affective Disorder or Psychotic Mood Disorder or Affective Psychosis or schizotypal or delusional or Bipolar Disorder or Manic Depressive Psychosis or Bipolar Affective Psychosis or Bipolar Affective Disorder or Manic Depressive Psychoses or Bipolar Depression or "Post-Traumatic Stress Disorder" or Post Traumatic Stress Disorder or Traumatic Stress Disorder or Stress Disorder or acute stress reaction or grief or Anxiety Disorder or phobi* or agoraphobi* or panic disorder or GAD or obsessive compulsive disorder or OCD).ti,ab.

4. health care delivery/ or health care access/ or patient-reported outcome/ or health care quality/ or patient compliance/ or health care disparity/ or health service/ or patient attitude/
5. (facilitat* or enhanc* or enable* or opportunity* or encourag* or motivat* or promot* or influen* or barrier* or challenge* or block* or challeng* or constrain* or deter* or difficult* or discourag* or disincentive* or encumber* or encumbranc* or hinder* or hindrance* or impair* or impede* or impeding or impediment* or limit* or delay or obstruct* or problem or restrain* or restrict* or interfer* or perceive* or perception* or perspective* or view* or experience* or need* or attitude* or belie* or opinion* or quality or Implementat* or adoption or patient experience or uptake* or utilis* or utiliz* or (("use" or acceptance or acceptability or availability or accessibility or access or accessing or receipt or receive or received or receiving) and (health care or healthcare or patient care or health service* or primary care or visit or appointment)) or ((provide or providing or provis* or distribut* or deliver*) and (health care or healthcare or patient care or health service* or primary care or visit or appointment))).ti,ab.
6. (afghanistan or albania or algeria or american samoa or angola or "antigua and barbuda" or antigua or barbuda or argentina or armenia or armenian or aruba or azerbaijan or bahrain or bangladesh or barbados or republic of belarus or belarus or byelarus or belorussia or byelorussian or belize or british honduras or benin or dahomey or bhutan or bolivia or "bosnia and herzegovina" or bosnia or herzegovina or botswana or bechuanaland or brazil or brasil or bulgaria or burkina faso or burkina fasso or upper volta or burundi or urundi or cabo verde or cape verde or cambodia or kampuchea or khmer republic or cameroon or cameron or cameroun or central african republic or ubangi shari or chad or chile or china or colombia or comoros or comoro islands or iles comores or mayotte or democratic republic of the congo or democratic republic congo or congo or zaire or costa rica or "cote d'ivoire" or "cote d' ivoire" or cote divoire or cote d ivoire or ivory coast or croatia or cuba or cyprus or czech republic or czechoslovakia or djibouti or french somaliland or dominica or dominican republic or ecuador or egypt or united arab republic or el salvador or equatorial guinea or spanish guinea or eritrea or estonia or eswatini or swaziland or ethiopia or fiji or gabon or gabonese republic or gambia or "georgia (republic)" or georgian or ghana or gold coast or gibraltar or greece or grenada or guam or guatemala or guinea or guinea bissau or guyana or british guiana or haiti or hispaniola or honduras or hungary or india or indonesia or timor or iran or iraq or isle of man or jamaica or jordan or kazakhstan or kazakh or kenya or "democratic people's republic of korea" or republic of korea or north korea or south korea or korea or kosovo or kyrgyzstan or kirghizia or kirgizstan or kyrgyz republic or kirghiz or laos or lao pdr or "lao people's democratic republic" or latvia or lebanon or lebanese republic or lesotho or basutoland or liberia or libya or libyan arab jamahiriya or lithuania or macau or macao or republic of north macedonia or macedonia or madagascar or malagasy republic or malawi or nyasaland or malaysia or malay federation or malaya federation or maldives or indian ocean islands or indian ocean or mali or malta or micronesia or federated states of micronesia or kiribati or marshall islands or nauru or northern mariana islands or palau or tuvalu or mauritania or mauritius or mexico or moldova or moldovian or mongolia or montenegro or "montenegro (republic)" or morocco or ifni or mozambique or portuguese east africa or myanmar or burma or namibia or nepal or netherlands antilles or nicaragua or niger or nigeria or oman or muscat or pakistan or panama or papua new guinea or new guinea or paraguay or peru or

philippines or philipines or phillipines or phillippines or poland or "polish people's republic" or portugal or portuguese republic or puerto rico or romania or russia or russian federation or ussr or soviet union or union of soviet socialist republics or rwanda or ruanda or samoa or pacific islands or polynesia or samoan islands or navigator island or navigator islands or "sao tome and principe" or saudi arabia or senegal or serbia or seychelles or sierra leone or slovakia or slovak republic or slovenia or melanesia or solomon island or solomon islands or norfolk island or norfolk islands or somalia or south africa or south sudan or sri lanka or ceylon or "saint kitts and nevis" or "st. kitts and nevis" or saint lucia or "st. lucia" or "saint vincent and the grenadines" or saint vincent or "st. vincent" or grenadines or sudan or suriname or surinam or dutch guiana or netherlands guiana or syria or syrian arab republic or tajikistan or tadjikistan or tadzhikistan or tadzhik or tanzania or tanganyika or thailand or siam or timor leste or east timor or togo or togolese republic or tonga or "trinidad and tobago" or trinidad or tobago or tunisia or "turkey (republic)" or turkey or turkmenistan or turkmen or uganda or ukraine or uruguay or uzbekistan or uzbek or vanuatu or new hebrides or venezuela or vietnam or viet nam or middle east or west bank or gaza or palestine or yemen or yugoslavia or zambia or zimbabwe or northern rhodesia or global south or africa south of the sahara or "sub saharan africa" or subsaharan africa or africa, central or central africa or africa, northern or north africa or northern africa or magreb or maghrib or sahara or africa, southern or southern africa or africa, eastern or east africa or eastern africa or africa, western or west africa or western africa or west indies or indian ocean islands or caribbean region or caribbean islands or caribbean or central america or latin america or "south and central america" or south america or asia, central or central asia or asia, northern or north asia or northern asia or asia, southeastern or southeastern asia or south eastern asia or southeast asia or south east asia or asia, western or western asia or europe, eastern or east europe or eastern europe or developing country or developing countries or developing nation? or developing population? or developing world or less developed countr* or less developed nation? or less developed population? or less developed world or lesser developed countr* or lesser developed nation? or lesser developed population? or lesser developed world or under developed countr* or under developed nation? or under developed population? or under developed world or underdeveloped countr* or underdeveloped nation? or underdeveloped population? or underdeveloped world or middle income countr* or middle income nation? or middle income population? or low income countr* or low income nation? or low income population? or lower income countr* or lower income nation? or lower income population? or underserved countr* or underserved nation? or underserved population? or underserved world or under served countr* or under served nation? or under served population? or under served world or deprived countr* or deprived nation? or deprived population? or deprived world or poor countr* or poor nation? or poor population? or poor world or poorer countr* or poorer nation? or poorer population? or poorer world or developing econom* or less developed econom* or lesser developed econom* or under developed econom* or underdeveloped econom* or middle income econom* or low income econom* or lower income econom* or low gdp or low gnp or low gross domestic or low gross national or lower gdp or lower gnp or lower gross domestic or lower gross national or lmic or lmics or third world or lami countr* or transitional countr* or emerging economies or emerging nation?).ti,ab.

7. (Qualitative research or Qualitative Method* or Qualitative or Focus group* or Interview* or Mixed Method*).ti,ab.

8. drug dependence treatment/ or Mental Health Services/
9. 1 or 2
10. 3 or 8
11. 4 or 5
12. 6 and 7 and 9 and 10 and 11
13. limit 12 to "reviews (best balance of sensitivity and specificity)"

PsychINFO

Domain	Search terms (MeSH term and/or Key words)
Population	Health Personnel/ or Caregivers/
	OR
	(service user* or consumer* or patient* or stakeholder* or user* or client* or carer* or caregiver* or parent* or family or relative* or guardian* or Health worker or Health care worker or Healthcare worker or Health Care Provider* or Healthcare Provider or Health professional or Health Care Professional or Healthcare Professional or Health staff or Medical staff or clinical staff or medical workforce or medical work force or health workforce or health work force).ti,ab.
	AND
Intervention<br>*Care for mental health conditions*	Preventive Mental Health Services/ or "substance use prevention"/ or "substance use treatment"/ or Mental Health Services/
	OR
	(Mental Health Service or Mental Healthcare or Mental Health Care or Mental Health System or Psychiatric Services or Psychiatric Care or Psychiatric Health Care or Psychiatric Healthcare or Mental Illness or Mental Health or Severe Mental Disorder or Common Mental Disorder or "Alcohol Use Disorder" or Alcohol Abuse or Alcohol-Related Disorder or Alcohol Related Disorder or Alcohol Addiction or Substance Disorder or Substance Abuse or Substance-Use Disorder or "Substance Use Disorder" or Opioid Abuse or Opiate

	Addiction or Opioid-Related Disorder or Opioid Related Disorder or Cannabis-Related Disorder or Cannabis Related Disorder or Cocaine Related Disorder or Cocaine Related Disorder or Cocaine Addiction or Amphetamine-Related Disorder or Amphetamine Related Disorder or Amphetamine Addiction or Heroin Dependence or Heroin Abuse or Heroin Addiction or Substance Induced Psychos or Substance-Induced Psychos or Depression or Depressive Disorder or Mood Disorder or Major Depressive Disorder or Attention Deficit Disorder or attention deficit hyperactivity disorder or ADHD or Conduct Disorder or Neurocognitive Disorder or Neurodevelopmental Disorder or Neurodevelopmental disabilit* or Developmental Disabilit* or Developmental Disorder or Autism Spectrum Disorder or Autistic Spectrum Disorder or Aspergers Disease or Aspergers Syndrome or learning disabilit* or intellectual disabilit* or intellectual development or mental retardation or hyperkinetic disorder or tic disorder or self-harm* or self-injur* or suicid* or Dementia or Alzheimer* or Parkinson* or Epilep* or seizure or Schizophrenia or Psychosis or Psychoses or Psychotic Disorder or Schizoaffective Disorder or Schizophreniform Disorder or Psychotic Affective Disorder or Psychotic Mood Disorder or Affective Psychosis or schizotypal or delusional or Bipolar Disorder or Manic Depressive Psychosis or Bipolar Affective Psychosis or Bipolar Affective Disorder or Manic Depressive Psychoses or Bipolar Depression or Post-Traumatic Stress Disorder or Post Traumatic Stress Disorder or Traumatic Stress Disorder or Stress Disorder or acute stress reaction or grief or Anxiety Disorder or phobi* or agoraphobi* or panic disorder or GAD or obsessive compulsive disorder or OCD).ab,ti.
	AND
Outcome<br>views and experience of care *uptake* and/or care *provision;* factors (barriers/facilitators) influencing service	Health Care Delivery/ or Client Attitudes/ or Health Care Access/ or Patient Reported Outcome Measures/ or Health Care Services/ or "Quality of Care"/ or "Quality of Services"/ or Treatment Compliance/ or Health Disparities/ or Mental Health Services/
	OR
	(facilitat* or enhanc* or enable* or opportunity* or encourag* or motivat* or promot* or influen* or barrier* or challenge* or block* or challeng* or constrain* or deter* or difficult* or discourag* or disincentive* or encumber* or encumbranc* or hinder* or hindrance* or impair* or impede* or impeding

uptake and/or care *provision*	or impediment* or limit* or delay or obstruct* or problem or restrain* or restrict* or interfer* or perceive* or perception* or perspective* or view* or experience* or need* or attitude* or belie* or opinion* or quality or Implementat* or adoption or patient experience or uptake* or utilis* or utiliz* or (("use" or acceptance or acceptability or availability or accessibility or access or accessing or receipt or receive or received or receiving) and (health care or healthcare or patient care or health service* or primary care or visit or appointment)) or ((provide or providing or provis* or distribut* or deliver*) and (health care or healthcare or patient care or health service* or primary care or visit or appointment))).ti,ab.
	AND
Context<br>*LMICs*	(afghanistan or albania or algeria or american samoa or angola or "antigua and barbuda" or antigua or barbuda or argentina or armenia or armenian or aruba or azerbaijan or bahrain or bangladesh or barbados or republic of belarus or belarus or byelarus or belorussia or byelorussian or belize or british honduras or benin or dahomey or bhutan or bolivia or "bosnia and herzegovina" or bosnia or herzegovina or botswana or bechuanaland or brazil or brasil or bulgaria or burkina faso or burkina fasso or upper volta or burundi or urundi or cabo verde or cape verde or cambodia or kampuchea or khmer republic or cameroon or cameron or cameroun or central african republic or ubangi shari or chad or chile or china or colombia or comoros or comoro islands or iles comores or mayotte or democratic republic of the congo or democratic republic congo or congo or zaire or costa rica or "cote d'ivoire" or "cote d' ivoire" or cote divoire or cote d ivoire or ivory coast or croatia or cuba or cyprus or czech republic or czechoslovakia or djibouti or french somaliland or dominica or dominican republic or ecuador or egypt or united arab republic or el salvador or equatorial guinea or spanish guinea or eritrea or estonia or eswatini or swaziland or ethiopia or fiji or gabon or gabonese republic or gambia or georgia or georgian or ghana or gold coast or gibraltar or greece or grenada or guam or guatemala or guinea or guinea bissau or guyana or british guiana or haiti or hispaniola or honduras or hungary or india or indonesia or timor or iran or iraq or isle of man or jamaica or jordan or kazakhstan or kazakh or kenya or "democratic people's republic of korea" or republic of korea or north korea or south korea or korea or kosovo or kyrgyzstan or kirghizia or kirgizstan or kyrgyz republic or kirghiz

	or laos or lao pdr or "lao people's democratic republic" or latvia or lebanon or lebanese republic or lesotho or basutoland or liberia or libya or libyan arab jamahiriya or lithuania or macau or macao or republic of north macedonia or macedonia or madagascar or malagasy republic or malawi or nyasaland or malaysia or malay federation or malaya federation or maldives or indian ocean islands or indian ocean or mali or malta or micronesia or federated states of micronesia or kiribati or marshall islands or nauru or northern mariana islands or palau or tuvalu or mauritania or mauritius or mexico or moldova or moldovian or mongolia or montenegro or morocco or ifni or mozambique or portuguese east africa or myanmar or burma or namibia or nepal or netherlands antilles or nicaragua or niger or nigeria or oman or muscat or pakistan or panama or papua new guinea or new guinea or paraguay or peru or philippines or philipines or phillipines or phillippines or poland or "polish people's republic" or portugal or portuguese republic or puerto rico or romania or russia or russian federation or ussr or soviet union or union of soviet socialist republics or rwanda or ruanda or samoa or pacific islands or polynesia or samoan islands or navigator island or navigator islands or "sao tome and principe" or saudi arabia or senegal or serbia or seychelles or sierra leone or slovakia or slovak republic or slovenia or melanesia or solomon island or solomon islands or norfolk island or norfolk islands or somalia or south africa or south sudan or sri lanka or ceylon or "saint kitts and nevis" or "st. kitts and nevis" or saint lucia or "st. lucia" or "saint vincent and the grenadines" or saint vincent or "st. vincent" or grenadines or sudan or suriname or surinam or dutch guiana or netherlands guiana or syria or syrian arab republic or tajikistan or tadjikistan or tadzhikistan or tadzhik or tanzania or tanganyika or thailand or siam or timor leste or east timor or togo or togolese republic or tonga or "trinidad and tobago" or trinidad or tobago or tunisia or turkey or turkmenistan or turkmen or uganda or ukraine or uruguay or uzbekistan or uzbek or vanuatu or new hebrides or venezuela or vietnam or viet nam or middle east or west bank or gaza or palestine or yemen or yugoslavia or zambia or zimbabwe or northern rhodesia or global south or africa south of the sahara or sub-saharan africa or subsaharan africa or africa, central or central africa or africa, northern or north africa or northern africa or magreb or maghrib or sahara or africa, southern or southern africa or africa, eastern or east africa or eastern africa or africa, western or west africa or western africa or west indies or indian ocean islands or caribbean or central america or latin

	america or "south and central america" or south america or asia, central or central asia or asia, northern or north asia or northern asia or asia, southeastern or southeastern asia or south eastern asia or southeast asia or south east asia or asia, western or western asia or europe, eastern or east europe or eastern europe or developing country or developing countries or developing nation* or developing population* or developing world or less developed countr* or less developed nation* or less developed population* or less developed world or lesser developed countr* or lesser developed nation* or lesser developed population* or lesser developed world or under developed countr* or under developed nation* or under developed population* or under developed world or underdeveloped countr* or underdeveloped nation* or underdeveloped population* or underdeveloped world or middle income countr* or middle income nation* or middle income population* or low income countr* or low income nation* or low income population* or lower income countr* or lower income nation* or lower income population* or underserved countr* or underserved nation* or underserved population* or underserved world or under served countr* or under served nation* or under served population* or under served world or deprived countr* or deprived nation* or deprived population* or deprived world or poor countr* or poor nation* or poor population* or poor world or poorer countr* or poorer nation* or poorer population* or poorer world or developing econom* or less developed econom* or lesser developed econom* or under developed econom* or underdeveloped econom* or middle income econom* or low income econom* or lower income econom* or low gdp or low gnp or low gross domestic or low gross national or lower gdp or lower gnp or lower gross domestic or lower gross national or lmic or lmics or third world or lami countr* or transitional countr* or emerging economies or emerging nation*).ti,ab.
	AND
Research type	(Qualitative research or Qualitative Method* or Qualitative or Focus group* or Interview* or Mixed Method*).ti,ab.

Search copied from PsychInfo on 07.03.2022

1. Health Personnel/ or Caregivers/
2. (service user* or consumer* or patient* or stakeholder* or user* or client* or carer* or caregiver* or parent* or family or relative* or guardian* or Health worker or Health care worker or Healthcare worker or Health Care Provider* or Healthcare Provider or Health professional or Health Care Professional or Healthcare Professional or Health staff or Medical staff or clinical staff or medical workforce or medical work force or health workforce or health work force).ti,ab.
3. 1 or 2
4. Preventive Mental Health Services/ or "substance use prevention"/ or "substance use treatment"/ or Mental Health Services/
5. (Mental Health Service or Mental Healthcare or Mental Health Care or Mental Health System or Psychiatric Services or Psychiatric Care or Psychiatric Health Care or Psychiatric Healthcare or Mental Illness or Mental Health or Severe Mental Disorder or Common Mental Disorder or "Alcohol Use Disorder" or Alcohol Abuse or Alcohol-Related Disorder or Alcohol Related Disorder or Alcohol Addiction or Substance Disorder or Substance Abuse or Substance-Use Disorder or "Substance Use Disorder" or Opioid Abuse or Opiate Addiction or Opioid-Related Disorder or Opioid Related Disorder or Cannabis-Related Disorder or Cannabis Related Disorder or Cocaine Related Disorder or Cocaine Related Disorder or Cocaine Addiction or Amphetamine-Related Disorder or Amphetamine Related Disorder or Amphetamine Addiction or Heroin Dependence or Heroin Abuse or Heroin Addiction or Substance Induced Psychos or Substance-Induced Psychos or Depression or Depressive Disorder or Mood Disorder or Major Depressive Disorder or Attention Deficit Disorder or attention deficit hyperactivity disorder or ADHD or Conduct Disorder or Neurocognitive Disorder or Neurodevelopmental Disorder or Neurodevelopmental disabilit* or Developmental Disabilit* or Developmental Disorder or Autism Spectrum Disorder or Autistic Spectrum Disorder or Aspergers Disease or Aspergers Syndrome or learning disabilit* or intellectual disabilit* or intellectual development or mental retardation or hyperkinetic disorder or tic disorder or self-harm* or self-injur* or suicid* or Dementia or Alzheimer* or Parkinson* or Epilep* or seizure or Schizophrenia or Psychosis or Psychoses or Psychotic Disorder or Schizoaffective Disorder or Schizophreniform Disorder or Psychotic Affective Disorder or Psychotic Mood Disorder or Affective Psychosis or schizotypal or delusional or Bipolar Disorder or Manic Depressive Psychosis or Bipolar Affective Psychosis or Bipolar Affective Disorder or Manic Depressive Psychoses or Bipolar Depression or Post-Traumatic Stress Disorder or Post Traumatic Stress Disorder or Traumatic Stress Disorder or Stress Disorder or acute stress reaction or grief or Anxiety Disorder or phobi* or agoraphobi* or panic disorder or GAD or obsessive compulsive disorder or OCD).ab,ti.
6. Health Care Delivery/ or Client Attitudes/ or Health Care Access/ or Patient Reported Outcome Measures/ or Health Care Services/ or "Quality of Care"/ or "Quality of Services"/ or Treatment Compliance/ or Health Disparities/ or Mental Health Services/

7. (facilitat* or enhanc* or enable* or opportunity* or encourag* or motivat* or promot* or influen* or barrier* or challenge* or block* or challeng* or constrain* or deter* or difficult* or discourag* or disincentive* or encumber* or encumbranc* or hinder* or hindrance* or impair* or impede* or impeding or impediment* or limit* or delay or obstruct* or problem or restrain* or restrict* or interfer* or perceive* or perception* or perspective* or view* or experience* or need* or attitude* or belie* or opinion* or quality or Implementat* or adoption or patient experience or uptake* or utilis* or utiliz* or (("use" or acceptance or acceptability or availability or accessibility or access or accessing or receipt or receive or received or receiving) and (health care or healthcare or patient care or health service* or primary care or visit or appointment)) or ((provide or providing or provis* or distribut* or deliver*) and (health care or healthcare or patient care or health service* or primary care or visit or appointment))).ti,ab.
8. (afghanistan or albania or algeria or american samoa or angola or "antigua and barbuda" or antigua or barbuda or argentina or armenia or armenian or aruba or azerbaijan or bahrain or bangladesh or barbados or republic of belarus or belarus or byelarus or belorussia or byelorussian or belize or british honduras or benin or dahomey or bhutan or bolivia or "bosnia and herzegovina" or bosnia or herzegovina or botswana or bechuanaland or brazil or brasil or bulgaria or burkina faso or burkina fasso or upper volta or burundi or urundi or cabo verde or cape verde or cambodia or kampuchea or khmer republic or cameroon or cameron or cameroun or central african republic or ubangi shari or chad or chile or china or colombia or comoros or comoro islands or iles comores or mayotte or democratic republic of the congo or democratic republic congo or congo or zaire or costa rica or "cote d’ivoire" or "cote d’ ivoire" or cote divoire or cote d ivoire or ivory coast or croatia or cuba or cyprus or czech republic or czechoslovakia or djibouti or french somaliland or dominica or dominican republic or ecuador or egypt or united arab republic or el salvador or equatorial guinea or spanish guinea or eritrea or estonia or eswatini or swaziland or ethiopia or fiji or gabon or gabonese republic or gambia or georgia or georgian or ghana or gold coast or gibraltar or greece or grenada or guam or guatemala or guinea or guinea bissau or guyana or british guiana or haiti or hispaniola or honduras or hungary or india or indonesia or timor or iran or iraq or isle of man or jamaica or jordan or kazakhstan or kazakh or kenya or "democratic people’s republic of korea" or republic of korea or north korea or south korea or korea or kosovo or kyrgyzstan or kirghizia or kirgizstan or kyrgyz republic or kirghiz or laos or lao pdr or "lao people's democratic republic" or latvia or lebanon or lebanese republic or lesotho or basutoland or liberia or libya or libyan arab jamahiriya or lithuania or macau or macao or republic of north macedonia or macedonia or madagascar or malagasy republic or malawi or nyasaland or malaysia or malay federation or malaya federation or maldives or indian ocean islands or indian ocean or mali or malta or micronesia or federated states of micronesia or kiribati or marshall islands or nauru or northern mariana islands or palau or tuvalu or mauritania or mauritius or mexico or moldova or moldovian or mongolia or montenegro or morocco or ifni or mozambique or portuguese east africa or myanmar or burma or namibia or nepal or netherlands antilles or nicaragua or niger or nigeria or oman or muscat or pakistan or panama or papua new guinea or new guinea or paraguay or peru or philippines or

philipines or phillipines or phillippines or poland or "polish people's republic" or portugal or portuguese republic or puerto rico or romania or russia or russian federation or ussr or soviet union or union of soviet socialist republics or rwanda or ruanda or samoa or pacific islands or polynesia or samoan islands or navigator island or navigator islands or "sao tome and principe" or saudi arabia or senegal or serbia or seychelles or sierra leone or slovakia or slovak republic or slovenia or melanesia or solomon island or solomon islands or norfolk island or norfolk islands or somalia or south africa or south sudan or sri lanka or ceylon or "saint kitts and nevis" or "st. kitts and nevis" or saint lucia or "st. lucia" or "saint vincent and the grenadines" or saint vincent or "st. vincent" or grenadines or sudan or suriname or surinam or dutch guiana or netherlands guiana or syria or syrian arab republic or tajikistan or tadjikistan or tadzhikistan or tadzhik or tanzania or tanganyika or thailand or siam or timor leste or east timor or togo or togolese republic or tonga or "trinidad and tobago" or trinidad or tobago or tunisia or turkey or turkmenistan or turkmen or uganda or ukraine or uruguay or uzbekistan or uzbek or vanuatu or new hebrides or venezuela or vietnam or viet nam or middle east or west bank or gaza or palestine or yemen or yugoslavia or zambia or zimbabwe or northern rhodesia or global south or africa south of the sahara or sub-saharan africa or subsaharan africa or africa, central or central africa or africa, northern or north africa or northern africa or magreb or maghrib or sahara or africa, southern or southern africa or africa, eastern or east africa or eastern africa or africa, western or west africa or western africa or west indies or indian ocean islands or caribbean or central america or latin america or "south and central america" or south america or asia, central or central asia or asia, northern or north asia or northern asia or asia, southeastern or southeastern asia or south eastern asia or southeast asia or south east asia or asia, western or western asia or europe, eastern or east europe or eastern europe or developing country or developing countries or developing nation* or developing population* or developing world or less developed countr* or less developed nation* or less developed population* or less developed world or lesser developed countr* or lesser developed nation* or lesser developed population* or lesser developed world or under developed countr* or under developed nation* or under developed population* or under developed world or underdeveloped countr* or underdeveloped nation* or underdeveloped population* or underdeveloped world or middle income countr* or middle income nation* or middle income population* or low income countr* or low income nation* or low income population* or lower income countr* or lower income nation* or lower income population* or underserved countr* or underserved nation* or underserved population* or underserved world or under served countr* or under served nation* or under served population* or under served world or deprived countr* or deprived nation* or deprived population* or deprived world or poor countr* or poor nation* or poor population* or poor world or poorer countr* or poorer nation* or poorer population* or poorer world or developing econom* or less developed econom* or lesser developed econom* or under developed econom* or underdeveloped econom* or middle income econom* or low income econom* or lower income econom* or low gdp or low gnp or low gross domestic or low gross national

or lower gdp or lower gnp or lower gross domestic or lower gross national or lmic or lmics or third world or lami countr* or transitional countr* or emerging economies or emerging nation*).ti,ab.

9. (Qualitative research or Qualitative Method* or Qualitative or Focus group* or Interview* or Mixed Method*).ti,ab.
10. 4 or 5
11. 6 or 7
12. 3 and 8 and 9 and 10 and 11
13. limit 12 to "reviews (best balance of sensitivity and specificity)"

CINAHL

Domain	Search terms (MH)
Population	health personnel or caregivers
	OR
	(service user* or consumer* or patient* or stakeholder* or user* or client* or carer* or caregiver* or parent* or family or relative* or guardian* or Health worker or Health care worker or Healthcare worker or Health Care Provider* or Healthcare Provider or Health professional or Health Care Professional or Healthcare Professional or Health staff or Medical staff or clinical staff or medical workforce or medical work force or health workforce or health work force)
	AND
Intervention<br>*Care for mental health conditions*	Preventive Health Care or mental health services
	OR
	(Mental Health Service or Mental Healthcare or Mental Health Care or Mental Health System or Psychiatric Services or Psychiatric Care or Psychiatric Health Care or Psychiatric Healthcare or Mental Illness or Mental Health or Severe Mental Disorder or Common Mental Disorder or "Alcohol Use Disorder" or Alcohol Abuse or Alcohol-Related Disorder or Alcohol Related Disorder or Alcohol Addiction or Substance Disorder or Substance Abuse or Substance-Use Disorder or "Substance Use Disorder" or Opioid Abuse or Opiate Addiction or Opioid-Related Disorder or Opioid Related Disorder or Cannabis-Related Disorder or Cannabis Related Disorder or Cocaine Related Disorder or Cocaine Related Disorder or Cocaine Addiction or Amphetamine-Related Disorder or Amphetamine Related Disorder or Amphetamine Addiction or Heroin Dependence or Heroin Abuse or Heroin Addiction or Substance Induced Psychos or Substance-Induced Psychos or Depression or Depressive Disorder or Mood Disorder or Major Depressive Disorder

	or Attention Deficit Disorder or attention deficit hyperactivity disorder or ADHD or Conduct Disorder or Neurocognitive Disorder or Neurodevelopmental Disorder or Neurodevelopmental disabilit* or Developmental Disabilit* or Developmental Disorder or Autism Spectrum Disorder or Autistic Spectrum Disorder or Aspergers Disease or Aspergers Syndrome or learning disabilit* or intellectual disabilit* or intellectual development or mental retardation or hyperkinetic disorder or tic disorder or self-harm* or self-injur* or suicid* or Dementia or Alzheimer* or Parkinson* or Epilep* or seizure or Schizophrenia or Psychosis or Psychoses or Psychotic Disorder or Schizoaffective Disorder or Schizophreniform Disorder or Psychotic Affective Disorder or Psychotic Mood Disorder or Affective Psychosis or schizotypal or delusional or Bipolar Disorder or Manic Depressive Psychosis or Bipolar Affective Psychosis or Bipolar Affective Disorder or Manic Depressive Psychoses or Bipolar Depression or Post-Traumatic Stress Disorder or Post Traumatic Stress Disorder or Traumatic Stress Disorder or Stress Disorder or acute stress reaction or grief or Anxiety Disorder or phobi* or agoraphobi* or panic disorder or GAD or obsessive compulsive disorder or OCD)
	AND
Outcome<br><br>views and experience of care *uptake* and/or care *provision;* factors (barriers/facilitators) influencing service *uptake* and/or care *provision*	"Health Services" or "Healthcare Disparities" or "Patient Compliance" or "Quality of Health Care" or "Patient-Reported Outcomes" or "Health Services Accessibility" or "Health Care Delivery" or "Patient Attitudes"
	OR
	(facilitat* or enhanc* or enable* or opportunity* or encourag* or motivat* or promot* or influen* or barrier* or challenge* or block* or challeng* or constrain* or deter* or difficult* or discourag* or disincentive* or encumber* or encumbranc* or hinder* or hindrance* or impair* or impede* or impeding or impediment* or limit* or delay or obstruct* or problem or restrain* or restrict* or interfer* or perceive* or perception* or perspective* or view* or experience* or need* or attitude* or belie* or opinion* or quality or Implementat* or adoption or patient experience or uptake* or utilis* or utiliz* or (("use" or acceptance or acceptability or availability or accessibility or access or accessing or receipt or receive or received or receiving) and (health care or healthcare or patient care or health service* or primary care or visit or appointment)) or ((provide or providing or provis* or distribut* or deliver*) and (health care or healthcare or patient care or health service* or primary care or visit or appointment)))
	AND
Context<br><br>*LMICs*	(afghanistan or albania or algeria or american samoa or angola or "antigua and barbuda" or antigua or barbuda or argentina or armenia or armenian or aruba or azerbaijan or bahrain or bangladesh or barbados or republic of belarus or belarus or byelarus or belorussia or byelorussian or belize or british honduras or benin or dahomey or bhutan or bolivia or "bosnia and herzegovina" or bosnia or herzegovina or botswana or bechuanaland or brazil or brasil or

	bulgaria or burkina faso or burkina fasso or upper volta or burundi or urundi or cabo verde or cape verde or cambodia or kampuchea or khmer republic or cameroon or cameron or cameroun or central african republic or ubangi shari or chad or chile or china or colombia or comoros or comoro islands or iles comores or mayotte or democratic republic of the congo or democratic republic congo or congo or zaire or costa rica or "cote d'ivoire" or "cote d' ivoire" or cote divoire or cote d ivoire or ivory coast or croatia or cuba or cyprus or czech republic or czechoslovakia or djibouti or french somaliland or dominica or dominican republic or ecuador or egypt or united arab republic or el salvador or equatorial guinea or spanish guinea or eritrea or estonia or eswatini or swaziland or ethiopia or fiji or gabon or gabonese republic or gambia or Georgia republic or georgian or ghana or gold coast or gibraltar or greece or grenada or guam or guatemala or guinea or guinea bissau or guyana or british guiana or haiti or hispaniola or honduras or hungary or india or indonesia or timor or iran or iraq or isle of man or jamaica or jordan or kazakhstan or kazakh or kenya or "democratic people's republic of korea" or republic of korea or north korea or south korea or korea or kosovo or kyrgyzstan or kirghizia or kirgizstan or kyrgyz republic or kirghiz or laos or lao pdr or "lao people's democratic republic" or latvia or lebanon or lebanese republic or lesotho or basutoland or liberia or libya or libyan arab jamahiriya or lithuania or macau or macao or republic of north macedonia or macedonia or madagascar or malagasy republic or malawi or nyasaland or malaysia or malay federation or malaya federation or maldives or indian ocean islands or indian ocean or mali or malta or micronesia or federated states of micronesia or kiribati or marshall islands or nauru or northern mariana islands or palau or tuvalu or mauritania or mauritius or mexico or moldova or moldovian or mongolia or montenegro or morocco or ifni or mozambique or portuguese east africa or myanmar or burma or namibia or nepal or netherlands antilles or nicaragua or niger or nigeria or oman or muscat or pakistan or panama or papua new guinea or new guinea or paraguay or peru or philippines or philipines or phillipines or phillippines or poland or "polish people's republic" or portugal or portuguese republic or puerto rico or romania or russia or russian federation or ussr or soviet union or union of soviet socialist republics or rwanda or ruanda or samoa or pacific islands or polynesia or samoan islands or navigator island or navigator islands or "sao tome and principe" or saudi arabia or senegal or serbia or seychelles or sierra leone or slovakia or slovak republic or slovenia or melanesia or solomon island or solomon islands or norfolk island or norfolk islands or somalia or south africa or south sudan or sri lanka or ceylon or "saint kitts and nevis" or "st. kitts and nevis" or saint lucia or "st. lucia" or "saint vincent and the grenadines" or saint vincent or "st. vincent" or grenadines or sudan or suriname or surinam or dutch guiana or netherlands guiana or syria or syrian arab republic or tajikistan or tadjikistan or tadzhikistan or tadzhik or tanzania or tanganyika or thailand or siam or timor leste or east timor or togo or togolese republic or

	tonga or "trinidad and tobago" or trinidad or tobago or tunisia or turkey or turkmenistan or turkmen or uganda or ukraine or uruguay or uzbekistan or uzbek or vanuatu or new hebrides or venezuela or vietnam or viet nam or middle east or west bank or gaza or palestine or yemen or yugoslavia or zambia or zimbabwe or northern rhodesia or global south or africa south of the sahara or sub-saharan africa or subsaharan africa or africa, central or central africa or africa, northern or north africa or northern africa or magreb or maghrib or sahara or africa, southern or southern africa or africa, eastern or east africa or eastern africa or africa, western or west africa or western africa or west indies or indian ocean islands or caribbean or central america or latin america or "south and central america" or south america or asia, central or central asia or asia, northern or north asia or northern asia or asia, southeastern or southeastern asia or south eastern asia or southeast asia or south east asia or asia, western or western asia or europe, eastern or east europe or eastern europe or developing country or developing countries or developing nation* or developing population* or developing world or less developed countr* or less developed nation* or less developed population* or less developed world or lesser developed countr* or lesser developed nation* or lesser developed population* or lesser developed world or under developed countr* or under developed nation* or under developed population* or under developed world or underdeveloped countr* or underdeveloped nation* or underdeveloped population* or underdeveloped world or middle income countr* or middle income nation* or middle income population* or low income countr* or low income nation* or low income population* or lower income countr* or lower income nation* or lower income population* or underserved countr* or underserved nation* or underserved population* or underserved world or under served countr* or under served nation* or under served population* or under served world or deprived countr* or deprived nation* or deprived population* or deprived world or poor countr* or poor nation* or poor population* or poor world or poorer countr* or poorer nation* or poorer population* or poorer world or developing econom* or less developed econom* or lesser developed econom* or under developed econom* or underdeveloped econom* or middle income econom* or low income econom* or lower income econom* or low gdp or low gnp or low gross domestic or low gross national or lower gdp or lower gnp or lower gross domestic or lower gross national or lmic or lmics or third world or lami countr* or transitional countr* or emerging economies or emerging nation*).ti,ab.
	AND
Research type	(Qualitative research or Qualitative Method* or Qualitative or Focus group* or Interview* or Mixed Method*)

Limit to Reviews (best balance)

AND

TI (“Qualitative research” OR “Qualitative Method*” OR Qualitative OR “Focus group*” OR Interview* OR “Mixed Method*”) OR AB (“Qualitative research” OR “Qualitative Method*” OR Qualitative OR “Focus group*” OR Interview* OR “Mixed Method*”)

AND

TI (afghanistan OR albania OR algeria OR "american samoa" OR angola OR "antigua and barbuda" OR antigua OR barbuda OR argentina OR armenia OR armenian OR aruba OR azerbaijan OR bahrain OR bangladesh OR barbados OR "republic of belarus" OR belarus OR byelarus OR belorussia OR byelorussian OR belize OR "british honduras" OR benin OR dahomey OR bhutan OR bolivia OR "bosnia and herzegovina" OR bosnia OR herzegovina OR botswana OR bechuanaland OR brazil OR brasil OR bulgaria OR "burkina faso" OR "burkina fasso" OR "upper volta" OR burundi OR urundi OR "cabo verde" OR "cape verde" OR cambodia OR kampuchea OR "khmer republic" OR cameroon OR cameron OR cameroun OR "central african republic" OR "ubangi shari" OR chad OR chile OR china OR colombia OR comoros OR "comoro islands" OR "iles comores" OR mayotte OR "democratic republic of the congo" OR "democratic republic congo" OR congo OR zaire OR "costa rica" OR "cote d’ivoire" OR "cote d’ ivoire" OR "cote divoire" OR "cote d ivoire" OR "ivory coast" OR croatia OR cuba OR cyprus OR "czech republic" OR czechoslovakia OR djibouti OR "french somaliland" OR dominica OR "dominican republic" OR ecuador OR egypt OR "united arab republic" OR "el salvador" OR "equatorial guinea" OR "spanish guinea" OR eritrea OR estonia OR eswatini OR swaziland OR ethiopia OR fiji OR gabon OR "gabonese republic" OR gambia OR "georgia republic" OR georgia OR georgian OR ghana OR "gold coast" OR gibraltar OR greece OR grenada OR

guam OR guatemala OR guinea OR "guinea bissau" OR guyana OR "british guiana" OR haiti OR hispaniola OR honduras OR hungary OR india OR indonesia OR timor OR iran OR iraq OR "isle of man" OR jamaica OR jordan OR kazakhstan OR kazakh OR kenya OR "democratic people’s republic of korea" OR "republic of korea" OR north korea OR south korea OR korea OR kosovo OR kyrgyzstan OR kirghizia OR kirgizstan OR "kyrgyz republic" OR kirghiz OR laos OR "lao pdr" OR "lao people's democratic republic" OR latvia OR lebanon OR "lebanese republic" OR lesotho OR basutoland OR liberia OR libya OR "libyan arab jamahiriya" OR lithuania OR macau OR macao OR "republic of north macedonia" OR macedonia OR madagascar OR "malagasy republic" OR malawi OR nyasaland OR malaysia OR "malay federation" OR "malaya federation" OR maldives OR "indian ocean islands" OR "indian ocean" OR mali OR malta OR micronesia OR "federated states of micronesia" OR kiribati OR "marshall islands" OR nauru OR "northern mariana islands" OR palau OR tuvalu OR mauritania OR mauritius OR mexico OR moldova OR moldovian OR mongolia OR montenegro OR morocco OR ifni OR mozambique OR "portuguese east africa" OR myanmar OR burma OR namibia OR nepal OR "netherlands antilles" OR nicaragua OR niger OR nigeria OR oman OR muscat OR pakistan OR panama OR "papua new guinea" OR paraguay OR peru OR philippines OR philipines OR phillipines OR phillippines OR poland OR "polish people's republic" OR portugal OR "portuguese republic" OR "puerto rico" OR romania OR russia OR "russian federation" OR ussr OR "soviet union" OR "union of soviet socialist republics" OR rwanda OR ruanda OR samoa OR "pacific islands" OR polynesia OR "samoan islands" OR "navigator island" OR "navigator islands" OR "sao tome and principe" OR "saudi arabia" OR senegal OR serbia OR seychelles OR "sierra leone" OR slovakia OR "slovak republic" OR slovenia OR melanesia OR "solomon island" OR "solomon islands" OR "norfolk island" OR "norfolk islands" OR somalia OR "south africa" OR "south sudan" OR "sri lanka" OR ceylon OR "saint kitts and nevis" OR "st. kitts and nevis" OR "saint lucia" OR "st. lucia" OR "saint vincent and the grenadines" OR "saint vincent" OR "st. vincent" OR grenadines OR sudan OR suriname OR surinam OR "dutch guiana" OR "netherlands guiana" OR syria OR "syrian arab republic" OR tajikistan OR tadjikistan OR tadzhikistan OR tadzhik OR tanzania OR tanganyika OR thailand OR siam OR "timor leste" OR "east timor" OR togo OR "togolese republic" OR tonga OR "trinidad and tobago" OR trinidad OR tobago OR tunisia OR turkey OR turkmenistan OR turkmen OR uganda OR ukraine OR uruguay OR uzbekistan OR uzbek OR vanuatu OR "new hebrides" OR venezuela OR vietnam OR "viet nam" OR "middle east" OR "west bank" OR gaza OR palestine OR yemen OR yugoslavia OR zambia OR zimbabwe OR "northern rhodesia" OR "global south" OR "africa south of the sahara" OR "sub saharan africa" OR "subsaharan africa" OR "africa, central" OR "central africa" OR "africa, northern" OR "north africa" OR "northern africa" OR magreb OR maghrib OR sahara OR "africa, southern" OR "southern africa" OR "africa, eastern" OR "east africa" OR "eastern africa" OR "africa, western" OR "west africa" OR "western africa" OR "west indies" OR "indian ocean islands" OR caribbean OR "central america" OR "latin america" OR "south and central america" OR "south america" OR "asia, central" OR "central asia" OR "asia, northern" OR "north asia" OR "northern asia" OR "asia, southeastern" OR "southeastern asia" OR "south eastern asia" OR "southeast asia" OR "south east asia" OR "asia, western" OR "western asia" OR "europe, eastern" OR "east europe" OR "eastern europe" OR "developing country" OR "developing countries" OR "developing nation" OR "developing nations" OR "developing population" OR "developing populations" OR "developing world" OR "less developed country" OR "less developed countries" OR "less developed nation" OR "less developed nations" OR "less developed population" OR "less developed populations" OR "less developed world" OR "lesser developed country" OR "lesser developed countries" OR "lesser developed nation" OR "lesser developed nations" OR "lesser developed population" OR "lesser developed populations" OR "lesser developed world" OR "under developed country" OR "under developed countries" OR "under developed nation" OR "under developed nations" OR "under developed population" OR "under developed populations" OR "under developed world" OR "underdeveloped country" OR "underdeveloped countries" OR "underdeveloped

nation" OR "underdeveloped nations" OR "underdeveloped population" OR "underdeveloped populations" OR "underdeveloped world" OR "middle income country" OR "middle income countries" OR "middle income nation" OR "middle income nations" OR "middle income population" OR "middle income populations" OR "low income country" OR "low income countries" OR "low income nation" OR "low income nations" OR "low income population" OR "low income populations" OR "lower income country" OR "lower income countries" OR "lower income nation" OR "lower income nations" OR "lower income population" OR "lower income populations" OR "underserved country" OR "underserved countries" OR "underserved nation" OR "underserved nations" OR "underserved population" OR "underserved populations" OR "underserved world" OR "under served country" OR "under served countries" OR "under served nation" OR "under served nations" OR "under served population" OR "under served populations" OR "under served world" OR "deprived country" OR "deprived countries" OR "deprived nation" OR "deprived nations" OR "deprived population" OR "deprived populations" OR "deprived world" OR "poor country" OR "poor countries" OR "poor nation" OR "poor nations" OR "poor population" OR "poor populations" OR "poor world" OR "poorer country" OR "poorer countries" OR "poorer nation" OR "poorer nations" OR "poorer population" OR "poorer populations" OR "poorer world" OR "developing economy" OR "developing economies" OR "less developed economy" OR "less developed economies" OR "lesser developed economy" OR "lesser developed economies" OR "under developed economy" OR "under developed economies" OR "underdeveloped economy" OR "underdeveloped economies" OR "middle income economy" OR "middle income economies" OR "low income economy" OR "low income economies" OR "lower income economy" OR "lower income economies" OR "low gdp" OR "low gnp" OR "low gross domestic" OR "low gross national" OR "lower gdp" OR "lower gnp" OR "lower gross domestic" OR "lower gross national" OR lmic OR lmics OR "third world" OR "lami country" OR "lami countries" OR "transitional country" OR "transitional countries" OR "emerging economies" OR "emerging nation" OR "emerging nations") OR AB (afghanistan OR albania OR algeria OR "american samoa" OR angola OR "antigua and barbuda" OR antigua OR barbuda OR argentina OR armenia OR armenian OR aruba OR azerbaijan OR bahrain OR bangladesh OR barbados OR "republic of belarus" OR belarus OR byelarus OR belorussia OR byelorussian OR belize OR "british honduras" OR benin OR dahomey OR bhutan OR bolivia OR "bosnia and herzegovina" OR bosnia OR herzegovina OR botswana OR bechuanaland OR brazil OR brasil OR bulgaria OR "burkina faso" OR "burkina fasso" OR "upper volta" OR burundi OR urundi OR "cabo verde" OR "cape verde" OR cambodia OR kampuchea OR "khmer republic" OR cameroon OR cameron OR cameroun OR "central african republic" OR "ubangi shari" OR chad OR chile OR china OR colombia OR comoros OR "comoro islands" OR "iles comores" OR mayotte OR "democratic republic of the congo" OR "democratic republic congo" OR congo OR zaire OR "costa rica" OR "cote d’ivoire" OR "cote d’ ivoire" OR "cote divoire" OR "cote d ivoire" OR "ivory coast" OR croatia OR cuba OR cyprus OR "czech republic" OR czechoslovakia OR djibouti OR "french somaliland" OR dominica OR "dominican republic" OR ecuador OR egypt OR "united arab republic" OR "el salvador" OR "equatorial guinea" OR "spanish guinea" OR eritrea OR estonia OR eswatini OR swaziland OR ethiopia OR fiji OR gabon OR "gabonese republic" OR gambia OR "georgia republic" OR georgia OR georgian OR ghana OR "gold coast" OR gibraltar OR greece OR grenada OR guam OR guatemala OR guinea OR "guinea bissau" OR guyana OR "british guiana" OR haiti OR hispaniola OR honduras OR hungary OR india OR indonesia OR timor OR iran OR iraq OR "isle of man" OR jamaica OR jordan OR kazakhstan OR kazakh OR kenya OR "democratic people’s republic of korea" OR "republic of korea" OR north korea OR south korea OR korea OR kosovo OR kyrgyzstan OR kirghizia OR kirgizstan OR "kyrgyz republic" OR kirghiz OR laos OR "lao pdr" OR "lao people's democratic republic" OR latvia OR lebanon OR "lebanese republic" OR lesotho OR basutoland OR liberia OR libya OR "libyan arab jamahiriya" OR lithuania OR macau OR macao OR "republic of north macedonia" OR macedonia OR madagascar OR "malagasy republic" OR malawi OR nyasaland OR malaysia OR "malay federation" OR "malaya federation"

OR maldives OR "indian ocean islands" OR "indian ocean" OR mali OR malta OR micronesia OR "federated states of micronesia" OR kiribati OR "marshall islands" OR nauru OR "northern mariana islands" OR palau OR tuvalu OR mauritania OR mauritius OR mexico OR moldova OR moldovian OR mongolia OR montenegro OR morocco OR ifni OR mozambique OR "portuguese east africa" OR myanmar OR burma OR namibia OR nepal OR "netherlands antilles" OR nicaragua OR niger OR nigeria OR oman OR muscat OR pakistan OR panama OR "papua new guinea" OR paraguay OR peru OR philippines OR philipines OR phillipines OR phillippines OR poland OR "polish people's republic" OR portugal OR "portuguese republic" OR "puerto rico" OR romania OR russia OR "russian federation" OR ussr OR "soviet union" OR "union of soviet socialist republics" OR rwanda OR ruanda OR samoa OR "pacific islands" OR polynesia OR "samoan islands" OR "navigator island" OR "navigator islands" OR "sao tome and principe" OR "saudi arabia" OR senegal OR serbia OR seychelles OR "sierra leone" OR slovakia OR "slovak republic" OR slovenia OR melanesia OR "solomon island" OR "solomon islands" OR "norfolk island" OR "norfolk islands" OR somalia OR "south africa" OR "south sudan" OR "sri lanka" OR ceylon OR "saint kitts and nevis" OR "st. kitts and nevis" OR "saint lucia" OR "st. lucia" OR "saint vincent and the grenadines" OR "saint vincent" OR "st. vincent" OR grenadines OR sudan OR suriname OR surinam OR "dutch guiana" OR "netherlands guiana" OR syria OR "syrian arab republic" OR tajikistan OR tadjikistan OR tadzhikistan OR tadzhik OR tanzania OR tanganyika OR thailand OR siam OR "timor leste" OR "east timor" OR togo OR "togolese republic" OR tonga OR "trinidad and tobago" OR trinidad OR tobago OR tunisia OR turkey OR turkmenistan OR turkmen OR uganda OR ukraine OR uruguay OR uzbekistan OR uzbek OR vanuatu OR "new hebrides" OR venezuela OR vietnam OR "viet nam" OR "middle east" OR "west bank" OR gaza OR palestine OR yemen OR yugoslavia OR zambia OR zimbabwe OR "northern rhodesia" OR "global south" OR "africa south of the sahara" OR "sub saharan africa" OR "subsaharan africa" OR "africa, central" OR "central africa" OR "africa, northern" OR "north africa" OR "northern africa" OR magreb OR maghrib OR sahara OR "africa, southern" OR "southern africa" OR "africa, eastern" OR "east africa" OR "eastern africa" OR "africa, western" OR "west africa" OR "western africa" OR "west indies" OR "indian ocean islands" OR caribbean OR "central america" OR "latin america" OR "south and central america" OR "south america" OR "asia, central" OR "central asia" OR "asia, northern" OR "north asia" OR "northern asia" OR "asia, southeastern" OR "southeastern asia" OR "south eastern asia" OR "southeast asia" OR "south east asia" OR "asia, western" OR "western asia" OR "europe, eastern" OR "east europe" OR "eastern europe" OR "developing country" OR "developing countries" OR "developing nation" OR "developing nations" OR "developing population" OR "developing populations" OR "developing world" OR "less developed country" OR "less developed countries" OR "less developed nation" OR "less developed nations" OR "less developed population" OR "less developed populations" OR "less developed world" OR "lesser developed country" OR "lesser developed countries" OR "lesser developed nation" OR "lesser developed nations" OR "lesser developed population" OR "lesser developed populations" OR "lesser developed world" OR "under developed country" OR "under developed countries" OR "under developed nation" OR "under developed nations" OR "under developed population" OR "under developed populations" OR "under developed world" OR "underdeveloped country" OR "underdeveloped countries" OR "underdeveloped nation" OR "underdeveloped nations" OR "underdeveloped population" OR "underdeveloped populations" OR "underdeveloped world" OR "middle income country" OR "middle income countries" OR "middle income nation" OR "middle income nations" OR "middle income population" OR "middle income populations" OR "low income country" OR "low income countries" OR "low income nation" OR "low income nations" OR "low income population" OR "low income populations" OR "lower income country" OR "lower income countries" OR "lower income nation" OR "lower income nations" OR "lower income population" OR "lower income populations" OR "underserved country" OR "underserved countries" OR "underserved nation" OR "underserved nations" OR "underserved population" OR "underserved populations" OR

"underserved world" OR "under served country" OR "under served countries" OR "under served nation" OR "under served nations" OR "under served population" OR "under served populations" OR "under served world" OR "deprived country" OR "deprived countries" OR "deprived nation" OR "deprived nations" OR "deprived population" OR "deprived populations" OR "deprived world" OR "poor country" OR "poor countries" OR "poor nation" OR "poor nations" OR "poor population" OR "poor populations" OR "poor world" OR "poorer country" OR "poorer countries" OR "poorer nation" OR "poorer nations" OR "poorer population" OR "poorer populations" OR "poorer world" OR "developing economy" OR "developing economies" OR "less developed economy" OR "less developed economies" OR "lesser developed economy" OR "lesser developed economies" OR "under developed economy" OR "under developed economies" OR "underdeveloped economy" OR "underdeveloped economies" OR "middle income economy" OR "middle income economies" OR "low income economy" OR "low income economies" OR "lower income economy" OR "lower income economies" OR "low gdp" OR "low gnp" OR "low gross domestic" OR "low gross national" OR "lower gdp" OR "lower gnp" OR "lower gross domestic" OR "lower gross national" OR lmic OR lmics OR "third world" OR "lami country" OR "lami countries" OR "transitional country" OR "transitional countries" OR "emerging economies" OR "emerging nation" OR "emerging nations")

AND

TI (facilitat* OR enhanc* OR enable* OR opportunity* OR encourag* OR motivat* OR promot* OR influen* OR barrier* OR challenge* OR block* OR challeng*

OR constrain* OR deter* OR difficult* OR discourag* OR disincentive* OR encumber* OR encumbranc* OR hinder* OR hindrance* OR impair* OR impede* OR impeding OR impediment* OR limit* OR delay OR obstruct* OR problem OR restrain* OR restrict* OR interfer* OR perceive* OR perception* OR perspective* OR view* OR experience* OR need* OR attitude* OR belie* OR opinion* OR quality OR Implementat* OR adoption OR “patient experience” OR uptake* OR utilis* OR utiliz* OR ((use OR acceptance OR acceptability OR availability OR accessibility OR access OR accessing OR receipt OR receive OR received OR receiving) AND (health care OR healthcare OR patient care OR “health service*” OR “primary care” OR visit OR appointment)) OR ((provide OR providing OR provis* OR distribut* OR deliver*) AND (“health care” OR healthcare OR “patient care” OR “health service*” OR “primary care” OR visit OR appointment))) OR AB (facilitat* OR enhanc* OR enable* OR opportunity* OR encourag* OR motivat* OR promot* OR influen* OR barrier* OR challenge* OR block* OR challeng* OR constrain* OR deter* OR difficult* OR discourag* OR disincentive* OR encumber* OR encumbranc* OR hinder* OR hindrance* OR impair* OR impede* OR impeding OR impediment* OR limit* OR delay OR obstruct* OR problem OR restrain* OR restrict* OR interfer* OR perceive* OR perception* OR perspective* OR view* OR experience* OR need* OR attitude* OR belie* OR opinion* OR quality OR Implementat* OR adoption OR “patient experience” OR uptake* OR utilis* OR utiliz* OR ((use OR acceptance OR acceptability OR availability OR accessibility OR access OR accessing OR receipt OR receive OR received OR receiving) AND (health care OR healthcare OR patient care OR “health service*” OR “primary care” OR visit OR

appointment)) OR ((provide OR providing OR provis* OR distribut* OR deliver*) AND (“health care” OR healthcare OR “patient care” OR “health service*” OR “primary care” OR visit OR appointment))) OR (MH "Health Services" or "Healthcare Disparities” or "Patient Compliance" or “Quality of Health Care" or “Patient-Reported Outcomes" or "Health Services Accessibility" or "Health Care Delivery" or "Patient Attitudes")

AND

TI (“Mental Health Service” OR “Mental Healthcare” OR “Mental Health Care” OR “Mental Health System” OR “Psychiatric Services” OR “Psychiatric Care” OR “Psychiatric Health Care” OR “Psychiatric Healthcare” OR “Mental Illness” OR “Mental Health” OR “Severe Mental Disorder” OR “Common Mental Disorder” OR “Alcohol Use Disorder” OR “Alcohol Abuse” OR “Alcohol-Related Disorder” OR “Alcohol Related Disorder” OR “Alcohol Addiction” OR “Substance Disorder” OR “Substance Abuse” OR “Substance-Use Disorder” OR “Substance Use Disorder” OR “Opioid Abuse” OR “Opiate Addiction” OR “Opioid-Related Disorder” OR “Opioid Related Disorder” OR “Cannabis-Related Disorder” OR “Cannabis Related Disorder” OR “Cocaine Related Disorder” OR “Cocaine Related Disorder” OR “Cocaine Addiction” OR “Amphetamine-Related Disorder” OR “Amphetamine Related Disorder” OR “Amphetamine Addiction” OR “Heroin Dependence” OR “Heroin Abuse” OR “Heroin Addiction” OR “Substance Induced Psychos” OR “Substance-Induced Psychos” OR Depression OR “Depressive Disorder” OR “Mood Disorder” OR “Major Depressive Disorder” OR “Attention Deficit Disorder” OR “attention deficit hyperactivity disorder” OR ADHD OR “Conduct Disorder” OR “Neurocognitive Disorder” OR “Neurodevelopmental Disorder” OR “Neurodevelopmental disabilit*” OR “Developmental Disabilit*” OR “Developmental Disorder” OR “Autism Spectrum Disorder” OR “Autistic Spectrum Disorder” OR “Aspergers Disease” OR “Aspergers Syndrome” OR “learning disabilit*” OR “intellectual disabilit*” OR “intellectual development” OR “mental retardation” OR “hyperkinetic disorder” OR “tic disorder” OR “self-harm*” OR “self-injur*” OR suicid* OR Dementia OR Alzheimer* OR Parkinson* OR Epilep* OR seizure OR Schizophrenia OR Psychosis OR Psychoses OR “Psychotic Disorder” OR “Schizoaffective Disorder” OR “Schizophreniform Disorder” OR “Psychotic Affective Disorder” OR “Psychotic Mood Disorder” OR “Affective Psychosis” OR schizotypal OR delusional OR “Bipolar Disorder” OR “Manic Depressive Psychosis” OR “Bipolar Affective Psychosis” OR “Bipolar Affective Disorder” OR “Manic Depressive Psychoses” OR “Bipolar Depression” OR “Post-Traumatic Stress Disorder” OR “Post Traumatic Stress Disorder” OR “Traumatic Stress Disorder” OR “Stress Disorder” OR “acute stress reaction” OR grief OR “Anxiety Disorder” OR phobi* OR agoraphobi* OR “panic disorder” OR GAD OR “obsessive compulsive disorder” OR OCD) OR AB (“Mental Health Service” OR “Mental Healthcare” OR “Mental Health Care” OR “Mental Health System” OR “Psychiatric Services” OR “Psychiatric Care” OR “Psychiatric Health Care” OR “Psychiatric Healthcare” OR “Mental Illness” OR “Mental Health” OR “Severe Mental Disorder” OR “Common Mental Disorder” OR “Alcohol Use Disorder” OR “Alcohol Abuse” OR “Alcohol-Related Disorder” OR “Alcohol Related Disorder” OR “Alcohol Addiction” OR “Substance Disorder” OR “Substance Abuse” OR “Substance-Use Disorder” OR “Substance Use Disorder” OR “Opioid Abuse” OR “Opiate Addiction” OR “Opioid-Related Disorder” OR “Opioid Related Disorder” OR “Cannabis-Related Disorder” OR “Cannabis Related Disorder” OR “Cocaine Related Disorder” OR “Cocaine Related Disorder” OR “Cocaine Addiction” OR “Amphetamine-Related Disorder” OR “Amphetamine Related Disorder” OR “Amphetamine Addiction” OR “Heroin Dependence” OR “Heroin

Abuse” OR “Heroin Addiction” OR “Substance Induced Psychos” OR “Substance-Induced Psychos” OR Depression OR “Depressive Disorder” OR “Mood Disorder” OR “Major Depressive Disorder” OR “Attention Deficit Disorder” OR “attention deficit hyperactivity disorder” OR ADHD OR “Conduct Disorder” OR “Neurocognitive Disorder” OR “Neurodevelopmental Disorder” OR “Neurodevelopmental disabilit*” OR “Developmental Disabilit*” OR “Developmental Disorder” OR “Autism Spectrum Disorder” OR “Autistic Spectrum Disorder” OR “Aspergers Disease” OR “Aspergers Syndrome” OR “learning disabilit*” OR “intellectual disabilit*” OR “intellectual development” OR “mental retardation” OR “hyperkinetic disorder” OR “tic disorder” OR “self-harm*” OR “self-injur*” OR suicid* OR Dementia OR Alzheimer* OR Parkinson* OR Epilep* OR seizure OR Schizophrenia OR Psychosis OR Psychoses OR “Psychotic Disorder” OR “Schizoaffective Disorder” OR “Schizophreniform Disorder” OR “Psychotic Affective Disorder” OR “Psychotic Mood Disorder” OR “Affective Psychosis” OR schizotypal OR delusional OR “Bipolar Disorder” OR “Manic Depressive Psychosis” OR “Bipolar Affective Psychosis” OR “Bipolar Affective Disorder” OR “Manic Depressive Psychoses” OR “Bipolar Depression” OR “Post-Traumatic Stress Disorder” OR “Post Traumatic Stress Disorder” OR “Traumatic Stress Disorder” OR “Stress Disorder” OR “acute stress reaction” OR grief OR “Anxiety Disorder” OR phobi* OR agoraphobi* OR “panic disorder” OR GAD OR “obsessive compulsive disorder” OR OCD) OR (MH Preventive Health Care or mental health services)

AND

TI (service user* OR consumer* OR patient* OR stakeholder* OR user* OR client* OR carer* OR caregiver* OR parent* OR family OR relative* OR guardian* OR Health worker OR Health care worker OR Healthcare worker OR Health Care Provider* OR Healthcare Provider OR Health professional OR Health Care Professional OR Healthcare Professional OR Health staff OR Medical staff OR clinical staff OR medical workforce OR medical work force OR health workforce OR health work force) OR AB (service user* OR consumer* OR patient* OR stakeholder* OR user* OR client* OR carer* OR caregiver* OR parent* OR family OR relative* OR guardian* OR Health worker OR Health care worker OR Healthcare worker OR Health Care Provider* OR Healthcare Provider OR Health professional OR Health Care Professional OR Healthcare Professional OR Health staff OR Medical staff OR clinical staff OR medical workforce OR medical work force OR health workforce OR health work force) OR (
MH health personnel or caregivers)

Scopus

Domain	Title and abstract
**Population**	service user* OR consumer* OR patient* OR stakeholder* OR user* OR client* OR carer* OR caregiver* OR parent* OR family OR relative* OR guardian* OR Health worker OR Health care worker OR Healthcare worker OR Health Care Provider* OR Healthcare Provider OR Health professional OR Health Care Professional OR Healthcare Professional OR Health staff OR Medical staff OR clinical staff OR medical workforce OR medical work force OR health workforce OR health work force
	AND
**Intervention**<br><br>*Care for mental health conditions*	“Mental Health Service” OR “Mental Healthcare” OR “Mental Health Care” OR “Mental Health System” OR “Psychiatric Services” OR “Psychiatric Care” OR “Psychiatric Health Care” OR “Psychiatric Healthcare” OR “Mental Illness” OR “Mental Health” OR “Severe Mental Disorder” OR “Common Mental Disorder” OR<br><br>“Alcohol Use Disorder” OR “Alcohol Abuse” OR “Alcohol-Related Disorder” OR “Alcohol Related Disorder” OR “Alcohol Addiction” OR<br><br>“Substance Disorder” OR “Substance Abuse” OR “Substance-Use Disorder” OR “Substance Use Disorder” OR<br><br>“Opioid Abuse” OR “Opiate Addiction” OR “Opioid-Related Disorder” OR “Opioid Related Disorder” OR “Cannabis-Related Disorder” OR “Cannabis Related Disorder” OR “Cocaine Related Disorder” OR “Cocaine Related Disorder” OR “Cocaine Addiction” OR “Amphetamine-Related Disorder” OR “Amphetamine Related Disorder” OR “Amphetamine Addiction” OR “Heroin Dependence” OR “Heroin Abuse” OR “Heroin Addiction” OR “Substance Induced Psychos” OR “Substance-Induced Psychos” OR<br><br>Depression OR “Depressive Disorder” OR “Mood Disorder” OR “Major Depressive Disorder” OR<br><br>“Attention Deficit Disorder” OR “attention deficit hyperactivity disorder” OR ADHD OR “Conduct Disorder” OR “Neurocognitive Disorder” OR “Neurodevelopmental Disorder” OR “Neurodevelopmental disabilit*” OR “Developmental Disabilit*” OR “Developmental Disorder” OR<br><br>“Autism Spectrum Disorder” OR “Autistic Spectrum Disorder” OR “Aspergers Disease” OR “Aspergers Syndrome” OR<br><br>“learning disabilit*” OR “intellectual disabilit*” OR “intellectual development” OR “mental retardation” OR “hyperkinetic disorder” OR “tic disorder” OR<br><br>“self-harm*” OR “self-injur*” OR suicid* OR

	Dementia OR Alzheimer* OR Parkinson* OR<br><br>Epilep* OR seizure OR<br><br>Schizophrenia OR Psychosis OR Psychoses OR "Psychotic Disorder" OR "Schizoaffective Disorder" OR "Schizophreniform Disorder" OR "Psychotic Affective Disorder" OR "Psychotic Mood Disorder" OR "Affective Psychosis" OR schizotypal OR delusional OR<br><br>"Bipolar Disorder" OR "Manic Depressive Psychosis" OR "Bipolar Affective Psychosis" OR "Bipolar Affective Disorder" OR "Manic Depressive Psychoses" OR "Bipolar Depression" OR "Post-Traumatic Stress Disorder" OR "Post Traumatic Stress Disorder" OR "Traumatic Stress Disorder" OR "Stress Disorder" OR "acute stress reaction" OR grief OR<br><br>"Anxiety Disorder" OR phobi* OR agoraphobi* OR "panic disorder" OR GAD OR "obsessive compulsive disorder" OR OCD
	AND
**Outcome**<br><br>views and experience of care *uptake* and/or care *provision;* factors (barriers/facilitators) influencing service *uptake* and/or care *provision*	facilitat* OR enhanc* OR enable* OR opportunity* OR encourag* OR motivat* OR promot* OR influen* OR barrier* OR challenge* OR block* OR challeng* OR constrain* OR deter* OR difficult* OR discourag* OR disincentive* OR encumber* OR encumbranc* OR hinder* OR hindrance* OR impair* OR impede* OR impeding OR impediment* OR limit* OR delay OR obstruct* OR problem OR restrain* OR restrict* OR interfer* OR perceive* OR perception* OR perspective* OR view* OR experience* OR need* OR attitude* OR belie* OR opinion* OR quality OR Implementat* OR adoption OR "patient experience" OR uptake* OR utilis* OR utiliz* OR ((use OR acceptance OR acceptability OR availability OR accessibility OR access OR accessing OR receipt OR receive OR received OR receiving) AND (health care OR healthcare OR patient care OR "health service*" OR "primary care" OR visit OR appointment)) OR ((provide OR providing OR provis* OR distribut* OR deliver*) AND ("health care" OR healthcare OR "patient care" OR "health service*" OR "primary care" OR visit OR appointment))
	AND
Context<br><br>LMICs	afghanistan OR albania OR algeria OR "american samoa" OR angola OR "antigua and barbuda" OR antigua OR barbuda OR argentina OR armenia OR armenian OR aruba OR azerbaijan OR bahrain OR bangladesh OR barbados OR "republic of belarus" OR belarus OR byelarus OR belorussia OR byelorussian OR belize OR "british honduras" OR benin OR dahomey OR bhutan OR bolivia OR "bosnia and herzegovina" OR bosnia

	OR herzegovina OR botswana OR bechuanaland OR brazil OR brasil OR bulgaria OR "burkina faso" OR "burkina fasso" OR "upper volta" OR burundi OR urundi OR "cabo verde" OR "cape verde" OR cambodia OR kampuchea OR "khmer republic" OR cameroon OR cameron OR cameroun OR "central african republic" OR "ubangi shari" OR chad OR chile OR china OR colombia OR comoros OR "comoro islands" OR "iles comores" OR mayotte OR "democratic republic of the congo" OR "democratic republic congo" OR congo OR zaire OR "costa rica" OR "cote d'ivoire" OR "cote d' ivoire" OR "cote divoire" OR "cote d ivoire" OR "ivory coast" OR croatia OR cuba OR cyprus OR "czech republic" OR czechoslovakia OR djibouti OR "french somaliland" OR dominica OR "dominican republic" OR ecuador OR egypt OR "united arab republic" OR "el salvador" OR "equatorial guinea" OR "spanish guinea" OR eritrea OR estonia OR eswatini OR swaziland OR ethiopia OR fiji OR gabon OR "gabonese republic" OR gambia OR "georgia (republic)" OR georgia OR georgian OR ghana OR "gold coast" OR gibraltar OR greece OR grenada OR guam OR guatemala OR guinea OR "guinea bissau" OR guyana OR "british guiana" OR haiti OR hispaniola OR honduras OR hungary OR india OR indonesia OR timor OR iran OR iraq OR "isle of man" OR jamaica OR jordan OR kazakhstan OR kazakh OR kenya OR "democratic people's republic of korea" OR "republic of korea" OR north korea OR south korea OR korea OR kosovo OR kyrgyzstan OR kirghizia OR kirgizstan OR "kyrgyz republic" OR kirghiz OR laos OR "lao pdr" OR "lao people's democratic republic" OR latvia OR lebanon OR "lebanese republic" OR lesotho OR basutoland OR liberia OR libya OR "libyan arab jamahiriya" OR lithuania OR macau OR macao OR "republic of north macedonia" OR macedonia OR madagascar OR "malagasy republic" OR malawi OR nyasaland OR malaysia OR "malay federation" OR "malaya federation" OR maldives OR "indian ocean islands" OR "indian ocean" OR mali OR malta OR micronesia OR "federated states of micronesia" OR kiribati OR "marshall islands" OR nauru OR "northern mariana islands" OR palau OR tuvalu OR mauritania OR mauritius OR mexico OR moldova OR moldovian OR mongolia OR montenegro OR morocco OR ifni OR mozambique OR "portuguese east africa" OR myanmar OR burma OR namibia OR nepal OR "netherlands antilles" OR nicaragua OR niger OR nigeria OR oman OR muscat OR pakistan OR panama OR "papua new guinea" OR paraguay OR peru OR philippines OR philipines OR phillipines OR phillippines OR poland OR "polish people's republic" OR portugal OR "portuguese republic" OR "puerto rico" OR romania OR russia OR "russian federation" OR ussr OR "soviet union" OR "union of soviet socialist republics" OR rwanda OR ruanda OR samoa OR "pacific islands" OR polynesia OR "samoan islands" OR "navigator island" OR "navigator islands" OR "sao tome and principe" OR "saudi arabia" OR senegal OR serbia OR seychelles OR "sierra leone" OR slovakia OR "slovak republic" OR slovenia OR melanesia OR "solomon island" OR "solomon islands" OR "norfolk island" OR "norfolk islands" OR somalia OR "south africa" OR "south sudan" OR "sri lanka" OR ceylon OR "saint kitts and nevis" OR "st. kitts and nevis" OR "saint lucia" OR "st. lucia" OR "saint vincent and the grenadines" OR "saint vincent" OR "st.

	vincent" OR grenadines OR sudan OR suriname OR surinam OR "dutch guiana" OR "netherlands guiana" OR syria OR "syrian arab republic" OR tajikistan OR tadjikistan OR tadzhikistan OR tadzhik OR tanzania OR tanganyika OR thailand OR siam OR "timor leste" OR "east timor" OR togo OR "togolese republic" OR tonga OR "trinidad and tobago" OR trinidad OR tobago OR tunisia OR turkey OR turkmenistan OR turkmen OR uganda OR ukraine OR uruguay OR uzbekistan OR uzbek OR vanuatu OR "new hebrides" OR venezuela OR vietnam OR "viet nam" OR "middle east" OR "west bank" OR gaza OR palestine OR yemen OR yugoslavia OR zambia OR zimbabwe OR "northern rhodesia" OR "global south" OR "africa south of the sahara" OR "sub saharan africa" OR "subsaharan africa" OR "africa, central" OR "central africa" OR "africa, northern" OR "north africa" OR "northern africa" OR magreb OR maghrib OR sahara OR "africa, southern" OR "southern africa" OR "africa, eastern" OR "east africa" OR "eastern africa" OR "africa, western" OR "west africa" OR "western africa" OR "west indies" OR "indian ocean islands" OR caribbean OR "central america" OR "latin america" OR "south and central america" OR "south america" OR "asia, central" OR "central asia" OR "asia, northern" OR "north asia" OR "northern asia" OR "asia, southeastern" OR "southeastern asia" OR "south eastern asia" OR "southeast asia" OR "south east asia" OR "asia, western" OR "western asia" OR "europe, eastern" OR "east europe" OR "eastern europe" OR "developing country" OR "developing countries" OR "developing nation" OR "developing nations" OR "developing population" OR "developing populations" OR "developing world" OR "less developed country" OR "less developed countries" OR "less developed nation" OR "less developed nations" OR "less developed population" OR "less developed populations" OR "less developed world" OR "lesser developed country" OR "lesser developed countries" OR "lesser developed nation" OR "lesser developed nations" OR "lesser developed population" OR "lesser developed populations" OR "lesser developed world" OR "under developed country" OR "under developed countries" OR "under developed nation" OR "under developed nations" OR "under developed population" OR "under developed populations" OR "under developed world" OR "underdeveloped country" OR "underdeveloped countries" OR "underdeveloped nation" OR "underdeveloped nations" OR "underdeveloped population" OR "underdeveloped populations" OR "underdeveloped world" OR "middle income country" OR "middle income countries" OR "middle income nation" OR "middle income nations" OR "middle income population" OR "middle income populations" OR "low income country" OR "low income countries" OR "low income nation" OR "low income nations" OR "low income population" OR "low income populations" OR "lower income country" OR "lower income countries" OR "lower income nation" OR "lower income nations" OR "lower income population" OR "lower income populations" OR "underserved country" OR "underserved countries" OR "underserved nation" OR "underserved nations" OR "underserved population" OR "underserved populations" OR "underserved world" OR "under served country" OR "under served countries" OR "under served nation"

	OR "under served nations" OR "under served population" OR "under served populations" OR "under served world" OR "deprived country" OR "deprived countries" OR "deprived nation" OR "deprived nations" OR "deprived population" OR "deprived populations" OR "deprived world" OR "poor country" OR "poor countries" OR "poor nation" OR "poor nations" OR "poor population" OR "poor populations" OR "poor world" OR "poorer country" OR "poorer countries" OR "poorer nation" OR "poorer nations" OR "poorer population" OR "poorer populations" OR "poorer world" OR "developing economy" OR "developing economies" OR "less developed economy" OR "less developed economies" OR "lesser developed economy" OR "lesser developed economies" OR "under developed economy" OR "under developed economies" OR "underdeveloped economy" OR "underdeveloped economies" OR "middle income economy" OR "middle income economies" OR "low income economy" OR "low income economies" OR "lower income economy" OR "lower income economies" OR "low gdp" OR "low gnp" OR "low gross domestic" OR "low gross national" OR "lower gdp" OR "lower gnp" OR "lower gross domestic" OR "lower gross national" OR lmic OR lmics OR "third world" OR "lami country" OR "lami countries" OR "transitional country" OR "transitional countries" OR "emerging economies" OR "emerging nation" OR "emerging nations"
	AND
**Research type**	“Qualitative research” OR “Qualitative Method*” OR Qualitative OR “Focus group*” OR Interview* OR “Mixed Method*”

Search retrieved from SCOPUS on 07.03.22

(TITLE-ABS (*afghanistan* OR *albania* OR *algeria* OR *"american samoa"* OR *angola* OR *"antigua and barbuda"* OR *antigua* OR *barbuda* OR *argentina* OR *armenia* OR *armenian* OR *aruba* OR *azerbaijan* OR *bahrain* OR *bangladesh* OR *barbados* OR *"republic of belarus"* OR *belarus* OR *byelarus* OR *belorussia* OR *byelorussian* OR *belize* OR *"british honduras"* OR *benin* OR *dahomey* OR *bhutan* OR *bolivia* OR *"bosnia and herzegovina"* OR *bosnia* OR *herzegovina* OR *botswana* OR *bechuanaland* OR *brazil* OR *brasil* OR *bulgaria* OR *"burkina faso"* OR *"burkina fasso"* OR *"upper volta"* OR *burundi* OR *urundi* OR *"cabo verde"* OR *"cape verde"* OR *cambodia* OR *kampuchea* OR *"khmer*

republic" OR *cameroon* OR *cameron* OR *cameroun* OR *"central african republic"* OR *"ubangi shari"* OR *chad* OR *chile* OR *china* OR *colombia* OR *comoros* OR *"comoro islands"* OR *"iles comores"* OR *mayotte* OR *"democratic republic of the congo"* OR *"democratic republic congo"* OR *congo* OR *zaire* OR *"costa rica"* OR *"cote d'ivoire"* OR *"cote d' ivoire"* OR *"cote divoire"* OR *"cote d ivoire"* OR *"ivory coast"* OR *croatia* OR *cuba* OR *cyprus* OR *"czech republic"* OR *czechoslovakia* OR *djibouti* OR *"french somaliland"* OR *dominica* OR *"dominican republic"* OR *ecuador* OR *egypt* OR *"united arab republic"* OR *"el salvador"* OR *"equatorial guinea"* OR *"spanish guinea"* OR *eritrea* OR *estonia* OR *eswatini* OR *swaziland* OR *ethiopia* OR *fiji* OR *gabon* OR *"gabonese republic"* OR *gambia* OR *georgia* OR *georgian* OR *ghana* OR *"gold coast"* OR *gibraltar* OR *greece* OR *grenada* OR *guam* OR *guatemala* OR *guinea* OR *"guinea bissau"* OR *guyana* OR *"british guiana"* OR *haiti* OR *hispaniola* OR *honduras* OR *hungary* OR *india* OR *indonesia* OR *timor* OR *iran* OR *iraq* OR *"isle of man"* OR *jamaica* OR *jordan* OR *kazakhstan* OR *kazakh* OR *kenya* OR *"democratic people's republic of korea"* OR *"republic of korea"* OR *"north korea"* OR *"south korea"* OR *korea* OR *kosovo* OR *kyrgyzstan* OR *kirghizia* OR *kirgizstan* OR *"kyrgyz republic"* OR *kirghiz* OR *laos* OR *"lao pdr"* OR *"lao people's democratic republic"* OR *latvia* OR *lebanon* OR *"lebanese republic"* OR *lesotho* OR *basutoland* OR *liberia* OR *libya* OR *"libyan arab jamahiriya"* OR *lithuania* OR *macau* OR *macao* OR *"republic of north macedonia"* OR *macedonia* OR *madagascar* OR *"malagasy republic"* OR *malawi* OR *nyasaland* OR *malaysia* OR *"malay federation"* OR *"malaya federation"* OR *maldives* OR *"indian ocean islands"* OR *"indian ocean"* OR *mali* OR *malta* OR *micronesia* OR *"federated states of micronesia"* OR *kiribati* OR *"marshall islands"* OR *nauru* OR *"northern mariana islands"* OR *palau* OR *tuvalu* OR *mauritania* OR *mauritius* OR *mexico* OR *moldova* OR *moldovian* OR *mongolia* OR *montenegro* OR *morocco* OR *if ni* OR *mozambique* OR *"portuguese east africa"* OR *myanmar* OR *burma* OR *namibia* OR *nepal* OR *"netherlands antilles"* OR *nicaragua* OR *niger* OR *nigeria* OR *oman* OR *muscat* OR *pakistan* OR *panama* OR *"papua new guinea"* OR *paraguay* OR *peru* OR *philippines* OR *philipines* OR *phillipines* OR *phillippines* OR *poland* OR *"polish people's republic"* OR *portugal* OR *"portuguese republic"* OR *"puerto rico"* OR *romania* OR *russia* OR *"russian federation"* OR *ussr* OR *"soviet union"* OR *"union of soviet socialist republics"* OR *rwanda* OR *ruanda* OR *samoa* OR *"pacific islands"* OR *polynesia* OR *"samoan islands"* OR *"navigator island"* OR *"navigator islands"* OR *"sao tome and principe"* OR *"saudi arabia"* OR *senegal* OR *serbia* OR *seychelles* OR *"sierra leone"* OR *slovakia* OR *"slovak republic"* OR *slovenia* OR *melanesia* OR *"solomon island"* OR *"solomon islands"* OR *"norfolk island"* OR *"norfolk islands"* OR *somalia* OR *"south africa"* OR *"south sudan"* OR *"sri lanka"* OR *ceylon* OR *"saint kitts and nevis"* OR *"st. kitts and nevis"* OR *"saint lucia"* OR *"st. lucia"* OR *"saint vincent and the grenadines"* OR *"saint vincent"* OR *"st. vincent"* OR *grenadines* OR *sudan* OR *suriname* OR *surinam* OR *"dutch guiana"* OR *"netherlands guiana"* OR *syria* OR *"syrian arab republic"* OR *tajikistan* OR *tadjikistan* OR *tadzhikistan* OR *tadzhik* OR *tanzania* OR *tanganyika* OR *thailand* OR *siam* OR *"timor leste"* OR *"east timor"* OR *togo* OR *"togolese republic"* OR *tonga* OR *"trinidad and tobago"* OR *trinidad* OR *tobago* OR *tunisia* OR *turkey* OR *turkmenistan* OR *turkmen* OR *uganda* OR *ukraine* OR *uruguay* OR *uzbekistan* OR *uzbek* OR *vanuatu* OR *"new hebrides"* OR *venezuela* OR *vietnam* OR *"viet nam"* OR *"middle east"* OR *"west bank"* OR *gaza* OR *palestine* OR *yemen* OR *yugoslavia* OR *zambia* OR *zimbabwe* OR *"northern rhodesia"* OR *"global south"* OR *"africa south of the*

sahara" OR *"sub saharan africa"* OR *"subsaharan africa"* OR *"africa, central"* OR *"central africa"* OR *"africa, northern"* OR *"north africa"* OR *"northern africa"* OR *magreb* OR *maghrib* OR *sahara* OR *"africa, southern"* OR *"southern africa"* OR *"africa, eastern"* OR *"east africa"* OR *"eastern africa"* OR *"africa, western"* OR *"west africa"* OR *"western africa"* OR *"west indies"* OR *"indian ocean islands"* OR *caribbean* OR *"central america"* OR *"latin america"* OR *"south and central america"* OR *"south america"* OR *"asia, central"* OR *"central asia"* OR *"asia, northern"* OR *"north asia"* OR *"northern asia"* OR *"asia, southeastern"* OR *"southeastern asia"* OR *"south eastern asia"* OR *"southeast asia"* OR *"south east asia"* OR *"asia, western"* OR *"western asia"* OR *"europe, eastern"* OR *"east europe"* OR *"eastern europe"* OR *"developing country"* OR *"developing countries"* OR *"developing nation"* OR *"developing nations"* OR *"developing population"* OR *"developing populations"* OR *"developing world"* OR *"less developed country"* OR *"less developed countries"* OR *"less developed nation"* OR *"less developed nations"* OR *"less developed population"* OR *"less developed populations"* OR *"less developed world"* OR *"lesser developed country"* OR *"lesser developed countries"* OR *"lesser developed nation"* OR *"lesser developed nations"* OR *"lesser developed population"* OR *"lesser developed populations"* OR *"lesser developed world"* OR *"under developed country"* OR *"under developed countries"* OR *"under developed nation"* OR *"under developed nations"* OR *"under developed population"* OR *"under developed populations"* OR *"under developed world"* OR *"underdeveloped country"* OR *"underdeveloped countries"* OR *"underdeveloped nation"* OR *"underdeveloped nations"* OR *"underdeveloped population"* OR *"underdeveloped populations"* OR *"underdeveloped world"* OR *"middle income country"* OR *"middle income countries"* OR *"middle income nation"* OR *"middle income nations"* OR *"middle income population"* OR *"middle income populations"* OR *"low income country"* OR *"low income countries"* OR *"low income nation"* OR *"low income nations"* OR *"low income population"* OR *"low income populations"* OR *"lower income country"* OR *"lower income countries"* OR *"lower income nation"* OR *"lower income nations"* OR *"lower income population"* OR *"lower income populations"* OR *"underserved country"* OR *"underserved countries"* OR *"underserved nation"* OR *"underserved nations"* OR *"underserved population"* OR *"underserved populations"* OR *"underserved world"* OR *"under served country"* OR *"under served countries"* OR *"under served nation"* OR *"under served nations"* OR *"under served population"* OR *"under served populations"* OR *"under served world"* OR *"deprived country"* OR *"deprived countries"* OR *"deprived nation"* OR *"deprived nations"* OR *"deprived population"* OR *"deprived populations"* OR *"deprived world"* OR *"poor country"* OR *"poor countries"* OR *"poor nation"* OR *"poor nations"* OR *"poor population"* OR *"poor populations"* OR *"poor world"* OR *"poorer country"* OR *"poorer countries"* OR *"poorer nation"* OR *"poorer nations"* OR *"poorer population"* OR *"poorer populations"* OR *"poorer world"* OR *"developing economy"* OR *"developing economies"* OR *"less developed economy"* OR *"less developed economies"* OR *"lesser developed economy"* OR *"lesser developed economies"* OR *"under developed economy"* OR *"under developed economies"* OR *"underdeveloped economy"* OR *"underdeveloped economies"* OR *"middle income economy"* OR *"middle income economies"* OR *"low income economy"* OR *"low income economies"* OR *"lower income economy"* OR *"lower income economies"* OR *"low gdp"* OR *"low gnp"* OR *"low gross domestic"* OR *"low gross national"* OR *"lower gdp"* OR *"lower gnp"* OR *"lower gross domestic"* OR *"lower gross national"* OR *lmic* OR *lmics* OR *"third world"* OR *"lami country"* OR *"lami countries"* OR *"transitional country"* OR *"transitional countries"* OR *"emerging economies"* OR *"emerging nation"* OR *"emerging nations"*))

AND

((TITLE-ABS (*"Qualitative research"* OR *"Qualitative Method*"* OR *qualitative* OR *"Focus group*"* OR *interview** OR *"Mixed Method*"*))

AND

(TITLE-ABS-KEY (*"service user*"* OR *consumer** OR *patient** OR *stakeholder** OR *user** OR *client** OR *carer** OR *caregiver** OR *parent** OR *family* OR *relative** OR *guardian** OR *"Health worker"* OR *"Health care worker"* OR *"Healthcare worker"* OR *"Health Care Provider*"* OR *"Healthcare Provider"* OR *"Health professional"* OR *"Health Care Professional"* OR *"Healthcare Professional"* OR *"Health staff"* OR *"Medical staff"* OR *"clinical staff"* OR *"medical workforce"* OR *"medical work force"* OR *"health workforce"* OR *"health work force"*))

AND

(TITLE-ABS-KEY (*facilitat** OR *enhanc** OR *enable** OR *opportunity** OR *encourag** OR *motivat** OR *promot** OR *influen** OR *barrier** OR *challenge** OR *block** OR *challeng** OR *constrain** OR *deter** OR *difficult** OR *discourag** OR *disincentive** OR *encumber** OR *encumbranc** OR *hinder** OR *hindrance** OR *impair** OR *impede** OR *impeding* OR *impediment** OR *limit** OR *delay* OR *obstruct** OR *problem* OR *restrain** OR *restrict** OR *interfer** OR *perceive** OR *perception** OR *perspective** OR *view** OR *experience** OR *need** OR *attitude** OR *belie** OR *opinion** OR *quality* OR *implementat** OR *adoption* OR *"patient experience"* OR *uptake** OR *utilis** OR *utiliz** OR ((*use* OR *acceptance* OR *acceptability* OR *availability* OR *accessibility* OR *access* OR *accessing* OR *receipt* OR *receive* OR *received* OR *receiving*) AND (*health* AND *care* OR *healthcare* OR *patient* AND *care* OR *"health service*"* OR *"primary care"* OR *visit* OR *appointment*)) OR ((*provide* OR *providing* OR *provis** OR *distribut** OR *deliver**) AND (*"health care"* OR *healthcare* OR *"patient care"* OR *"health service*"* OR *"primary care"* OR *visit* OR *appointment*)))) AND (TITLE-ABS-KEY (*"Mental Health*

Service" OR *"Mental Healthcare"* OR *"Mental Health Care"* OR *"Mental Health System"* OR *"Psychiatric Services"* OR *"Psychiatric Care"* OR *"Psychiatric Health Care"* OR *"Psychiatric Healthcare"* OR *"Mental Illness"* OR *"Mental Health"* OR *"Severe Mental Disorder"* OR *"Common Mental Disorder"* OR *"Alcohol Use Disorder"* OR *"Alcohol Abuse"* OR *"Alcohol-Related Disorder"* OR *"Alcohol Related Disorder"* OR *"Alcohol Addiction"* OR *"Substance Disorder"* OR *"Substance Abuse"* OR *"Substance-Use Disorder"* OR *"Substance Use Disorder"* OR *"Opioid Abuse"* OR *"Opiate Addiction"* OR *"Opioid-Related Disorder"* OR *"Opioid Related Disorder"* OR *"Cannabis-Related Disorder"* OR *"Cannabis Related Disorder"* OR *"Cocaine Related Disorder"* OR *"Cocaine Related Disorder"* OR *"Cocaine Addiction"* OR *"Amphetamine-Related Disorder"* OR *"Amphetamine Related Disorder"* OR *"Amphetamine Addiction"* OR *"Heroin Dependence"* OR *"Heroin Abuse"* OR *"Heroin Addiction"* OR *"Substance Induced Psychos"* OR *"Substance-Induced Psychos"* OR *depression* OR *"Depressive Disorder"* OR *"Mood Disorder"* OR *"Major Depressive Disorder"* OR *"Attention Deficit Disorder"* OR *"attention deficit hyperactivity disorder"* OR *adhd* OR *"Conduct Disorder"* OR *"Neurocognitive Disorder"* OR *"Neurodevelopmental Disorder"* OR *"Neurodevelopmental disabilit*"* OR *"Developmental Disabilit*"* OR *"Developmental Disorder"* OR *"Autism Spectrum Disorder"* OR *"Autistic Spectrum Disorder"* OR *"Aspergers Disease"* OR *"Aspergers Syndrome"* OR *"learning disabilit*"* OR *"intellectual disabilit*"* OR *"intellectual development"* OR *"mental retardation"* OR *"hyperkinetic disorder"* OR *"tic disorder"* OR *"self-harm*"* OR *"self-injur*"* OR *suicid** OR *dementia* OR *alzheimer** OR *parkinson** OR *epilep** OR *seizure* OR *schizophrenia* OR *psychosis* OR *psychoses* OR *"Psychotic Disorder"* OR *"Schizoaffective Disorder"* OR *"Schizophreniform Disorder"* OR *"Psychotic Affective Disorder"* OR *"Psychotic Mood Disorder"* OR *"Affective Psychosis"* OR *schizotypal* OR *delusional* OR *"Bipolar Disorder"* OR *"Manic Depressive Psychosis"* OR *"Bipolar Affective Psychosis"* OR *"Bipolar Affective Disorder"* OR *"Manic Depressive Psychoses"* OR *"Bipolar Depression"* OR *"Post-Traumatic Stress Disorder"* OR *"Post Traumatic Stress Disorder"* OR *"Traumatic Stress Disorder"* OR *"Stress Disorder"* OR *"acute stress reaction"* OR *grief* OR *"Anxiety Disorder"* OR *phobi** OR *agoraphobi** OR *"panic disorder"* OR *gad* OR *"obsessive compulsive disorder"* OR *ocd*)))

AND

(LIMIT-TO (DOCTYPE , *"re"*))

Global Index Medicus: African Index Medicus, Index Medicus for the Eastern Mediterranean Region, Index Medicus for the South-East Asian Region, Latin American and Caribbean Health Sciences Literature, and Western Pacific Region Index Medicus

CONNECTOR	Search string	Type of search
	"Caregivers" OR "Persons with Mental Disabilities" OR "Mentally Ill Persons" OR "Health Personnel"	Subject descriptor
OR	tw:(tw:((tw:(tw:(service user* OR consumer* OR patient* OR stakeholder* OR user* OR client*))) OR (tw:(tw:(carer* OR caregiver* OR parent* OR family OR relative* OR guardian*))) OR (tw:(tw:(health worker OR health care worker OR healthcare worker OR health care provider* OR healthcare provider OR health professional))) OR (tw:(tw:(health care professional OR healthcare professional OR health staff OR medical staff OR clinical staff))) OR (tw:(tw:(medical workforce OR medical work force OR health workforce OR health work force)))))	Title, abstract, subject
AND	"Mental Disorders"	Subject descriptor
OR	tw:((tw:(tw:(mental health service OR mental healthcare OR mental health care OR mental health system OR psychiatric services OR psychiatric care OR psychiatric health care OR psychiatric healthcare))) OR (tw:(tw:(mental illness OR mental health OR severe mental disorder OR common mental disorder))) OR (tw:(tw:(alcohol use disorder OR alcohol abuse OR alcohol-related disorder OR alcohol related disorder OR alcohol addiction))) OR (tw:(tw:(substance disorder OR substance abuse OR substance-use disorder OR substance use disorder))) OR (tw:(tw:(opioid abuse OR opiate addiction OR opioid-related disorder OR opioid related disorder))) OR (tw:(tw:(cannabis-related disorder OR cannabis related disorder))) OR (tw:(tw:(cocaine related disorder OR cocaine related disorder OR cocaine addiction))) OR (tw:(tw:(amphetamine-related disorder OR amphetamine related disorder OR amphetamine addiction))) OR (tw:(tw:(heroin dependence OR heroin abuse OR heroin addiction))) OR (tw:(tw:(depression OR depressive disorder OR mood disorder OR major depressive disorder))) OR (tw:(tw:(attention deficit disorder OR attention deficit hyperactivity disorder OR adhd OR conduct disorder))) OR (tw:(tw:(neurocognitive disorder OR neurodevelopmental disorder OR neurodevelopmental disabilit* OR developmental disabilit* OR developmental disorder))) OR (tw:(tw:(autism spectrum disorder OR autistic spectrum disorder OR aspergers disease OR aspergers syndrome))) OR (tw:(tw:(learning disabilit* OR intellectual disabilit* OR intellectual development OR mental retardation OR hyperkinetic disorder OR tic disorder))) OR (tw:(tw:(self-harm* OR self-injur* OR suicid*))) OR (tw:(tw:(dementia OR alzheimer* OR parkinson*))) OR (tw:(tw:(epilep* OR seizure))) OR (tw:(tw:(schizophrenia OR psychosis OR psychoses OR psychotic disorder))) OR (tw:(tw:(schizoaffective disorder OR schizophreniform disorder OR psychotic affective disorder OR psychotic mood disorder OR affective psychosis OR schizotypal OR delusional))) OR (tw:(tw:(bipolar disorder OR manic depressive psychosis OR bipolar affective psychosis OR bipolar affective disorder OR manic depressive psychoses OR bipolar depression))) OR (tw:(tw:(post-traumatic stress disorder OR post traumatic stress disorder OR traumatic stress disorder OR stress disorder OR	Title, abstract, subject

	acute stress reaction OR grief))) OR (tw:(tw:(anxiety disorder OR phobi* OR agoraphobi* OR "panic disorder" OR gad OR "obsessive compulsive disorder" OR ocd))))	
AND	"Health Services Accessibility" OR "Patient Acceptance of Health Care" OR "Delivery of Health Care" OR "Quality of Health Care"	Subject descriptor
OR	(tw:(tw:(facilitat* OR enhanc* OR enable* OR opportunity* OR encourag* OR motivat* OR promot* OR influen*))) OR (tw:(tw:(barrier* OR challenge* OR block* OR challeng* OR constrain* OR deter* OR difficult* OR discourag* OR disincentive* OR encumber* OR encumbranc* OR hinder* OR hindrance* OR impair* OR impede* OR impeding OR impediment* OR limit* OR delay OR obstruct* OR problem OR restrain* OR restrict* OR interfer*))) OR (tw:(tw:(perceive* OR perception* OR perspective* OR view* OR experience* OR need* OR attitude* OR belie* OR opinion*))) OR (tw:(tw:(quality OR implementat* OR adoption))) OR (tw:(tw:(patient experience OR uptake* OR utilis* OR utiliz*))) OR (tw:(tw:(((use OR acceptance OR acceptability OR availability OR accessibility OR access OR accessing OR receipt OR receive OR received OR receiving) AND (health care OR healthcare OR patient care OR health service* OR primary care OR visit OR appointment))))) OR (tw:(tw:(((provide OR providing OR provis* OR distribut* OR deliver*) AND (health care OR healthcare OR patient care OR health service* OR primary care OR visit OR appointment)))))	Title, abstract, subject
AND	(tw:(tw:(qualitative research OR qualitative method*))) OR (tw:(tw:(qualitative OR mixed method*))) OR (tw:(tw:(focus group* OR interview*)))	Title, abstract, subject

Resulting in this search:

tw:((mh:("Caregivers" OR "Persons with Mental Disabilities" OR "Mentally Ill Persons" OR "Health Personnel")) OR (tw:(tw:(tw:((tw:(tw:(service user* OR consumer* OR patient* OR stakeholder* OR user* OR client*))) OR (tw:(tw:(carer* OR caregiver* OR parent* OR family OR relative* OR guardian*))) OR (tw:(tw:(health worker OR health care worker OR healthcare worker OR health care provider* OR healthcare provider OR health professional))) OR (tw:(tw:(health care professional OR healthcare professional OR health staff OR medical staff OR clinical staff))) OR (tw:(tw:(medical workforce OR medical work force OR health workforce OR health work force)))))))

AND

(mh:("Mental Disorders")) OR (tw:(tw:((tw:(tw:(mental health service OR mental healthcare OR mental health care OR mental health system OR psychiatric services OR psychiatric care OR psychiatric health care OR psychiatric healthcare))) OR (tw:(tw:(mental illness OR mental health OR severe mental disorder

OR common mental disorder))) OR (tw:(tw:(alcohol use disorder OR alcohol abuse OR alcohol-related disorder OR alcohol related disorder OR alcohol addiction))) OR (tw:(tw:(substance disorder OR substance abuse OR substance-use disorder OR substance use disorder))) OR (tw:(tw:(opioid abuse OR opiate addiction OR opioid-related disorder OR opioid related disorder))) OR (tw:(tw:(cannabis-related disorder OR cannabis related disorder))) OR (tw:(tw:(cocaine related disorder OR cocaine related disorder OR cocaine addiction))) OR (tw:(tw:(amphetamine-related disorder OR amphetamine related disorder OR amphetamine addiction))) OR (tw:(tw:(heroin dependence OR heroin abuse OR heroin addiction))) OR (tw:(tw:(depression OR depressive disorder OR mood disorder OR major depressive disorder))) OR (tw:(tw:(attention deficit disorder OR attention deficit hyperactivity disorder OR adhd OR conduct disorder))) OR (tw:(tw:(neurocognitive disorder OR neurodevelopmental disorder OR neurodevelopmental disabilit* OR developmental disabilit* OR developmental disorder))) OR (tw:(tw:(autism spectrum disorder OR autistic spectrum disorder OR aspergers disease OR aspergers syndrome))) OR (tw:(tw:(learning disabilit* OR intellectual disabilit* OR intellectual development OR mental retardation OR hyperkinetic disorder OR tic disorder))) OR (tw:(tw:(self-harm* OR self-injur* OR suicid*))) OR (tw:(tw:(dementia OR alzheimer* OR parkinson*))) OR (tw:(tw:(epilep* OR seizure))) OR (tw:(tw:(schizophrenia OR psychosis OR psychoses OR psychotic disorder))) OR (tw:(tw:(schizoaffective disorder OR schizophreniform disorder OR psychotic affective disorder OR psychotic mood disorder OR affective psychosis OR schizotypal OR delusional))) OR (tw:(tw:(bipolar disorder OR manic depressive psychosis OR bipolar affective psychosis OR bipolar affective disorder OR manic depressive psychoses OR bipolar depression))) OR (tw:(tw:(post-traumatic stress disorder OR post traumatic stress disorder OR traumatic stress disorder OR stress disorder OR acute stress reaction OR grief))) OR (tw:(tw:(anxiety disorder OR phobi* OR agoraphobi* OR "panic disorder" OR gad OR "obsessive compulsive disorder" OR ocd))))))

AND

(mh:("Health Services Accessibility" OR "Patient Acceptance of Health Care" OR "Delivery of Health Care" OR "Quality of Health Care")) OR (tw:((tw:(tw:(facilitat* OR enhanc* OR enable* OR opportunity* OR encourag* OR motivat* OR promot* OR influen*))) OR (tw:(tw:(barrier* OR challenge* OR block* OR challeng* OR constrain* OR deter* OR difficult* OR discourag* OR disincentive* OR encumber* OR encumbranc* OR hinder* OR hindrance* OR impair* OR impede* OR impeding OR impediment* OR limit* OR delay OR obstruct* OR problem OR restrain* OR restrict* OR interfer*))) OR (tw:(tw:(perceive* OR perception* OR perspective* OR view* OR experience* OR need* OR attitude* OR belie* OR opinion*))) OR (tw:(tw:(quality OR implementat* OR adoption))) OR (tw:(tw:(patient experience OR uptake* OR utilis* OR utiliz*))) OR (tw:(tw:(((use OR acceptance OR acceptability OR availability OR accessibility OR access OR accessing OR receipt OR receive OR received OR receiving)

AND

(health care OR healthcare OR patient care OR health service* OR primary care OR visit OR appointment))))) OR (tw:(tw:(((provide OR providing OR provis* OR distribut* OR deliver*) AND (health care OR healthcare OR patient care OR health service* OR primary care OR visit OR appointment))))))) AND (tw:((tw:(tw:(qualitative research OR qualitative method*))) OR (tw:(tw:(qualitative OR mixed method*))) OR (tw:(tw:(focus group* OR interview*))))))

AND

(type_of_study:("systematic_reviews" OR "policy_brief" OR "sysrev_observational_studies"))

Repositories of systematic reviews protocols (PROSPERO, Open Science Framework (OSF), and Cochrane)

Key words used in searches –

(Mental health) AND (service user or health care worker) AND (experience or attitudes) AND (low and middle income countries)

Prospero (without lmic) – 108

Cochrane - 204

OSF – 117 (+ tags: (“systematic review”))
